# On the Three-Finger Protein Domain Fold and CD59-Like Proteins in *Schistosoma mansoni*

**DOI:** 10.1371/journal.pntd.0002482

**Published:** 2013-10-24

**Authors:** Leonardo P. Farias, Greice Krautz-Peterson, Cibele A. Tararam, Bogar O. Araujo-Montoya, Tatiana R. Fraga, Henrique K. Rofatto, Floriano P. Silva-Jr, Lourdes Isaac, Akram A. Da'dara, R. Alan Wilson, Charles B. Shoemaker, Luciana C. C. Leite

**Affiliations:** 1 Centro de Biotecnologia, Instituto Butantan, São Paulo, Brazil; 2 Department of Infectious Diseases and Global Health, Tufts University Cummings School of Veterinary Medicine, North Grafton, Massachusetts, United States of America; 3 Programa de Pós-Graduação Interunidades em Biotecnologia, Universidade de São Paulo, São Paulo, Brazil; 4 LBPP, Instituto Oswaldo Cruz/FioCruz, Manguinhos, Rio de Janeiro, Brazil; 5 Departamento de Imunologia, Universidade de São Paulo, São Paulo, Brazil; 6 Department of Biology, University of York, York, United Kingdom; McGill University, Canada

## Abstract

**Background:**

It is believed that schistosomes evade complement-mediated killing by expressing regulatory proteins on their surface. Recently, six homologues of human CD59, an important inhibitor of the complement system membrane attack complex, were identified in the schistosome genome. Therefore, it is important to investigate whether these molecules could act as CD59-like complement inhibitors in schistosomes as part of an immune evasion strategy.

**Methodology/Principal Findings:**

Herein, we describe the molecular characterization of seven putative SmCD59-like genes and attempt to address the putative biological function of two isoforms. Superimposition analysis of the 3D structure of hCD59 and schistosome sequences revealed that they contain the three-fingered protein domain (TFPD). However, the conserved amino acid residues involved in complement recognition in mammals could not be identified. Real-time RT-PCR and Western blot analysis determined that most of these genes are up-regulated in the transition from free-living cercaria to adult worm stage. Immunolocalization experiments and tegument preparations confirm that at least some of the SmCD59-like proteins are surface-localized; however, significant expression was also detected in internal tissues of adult worms. Finally, the involvement of two SmCD59 proteins in complement inhibition was evaluated by three different approaches: (i) a hemolytic assay using recombinant soluble forms expressed in *Pichia pastoris* and *E. coli*; (ii) complement-resistance of CHO cells expressing the respective membrane-anchored proteins; and (iii) the complement killing of schistosomula after gene suppression by RNAi. Our data indicated that these proteins are not involved in the regulation of complement activation.

**Conclusions:**

Our results suggest that this group of proteins belongs to the TFPD superfamily. Their expression is associated to intra-host stages, present in the tegument surface, and also in intra-parasite tissues. Three distinct approaches using SmCD59 proteins to inhibit complement strongly suggested that these proteins are not complement inhibitors and their function in schistosomes remains to be determined.

## Author Summary

Schistosomes are parasites that reside for many years in the blood stream, demanding efficient mechanisms of evading immune response effectors such as complement deposition. A group of genes similar to human CD59, an important complement inhibitor in mammals, were identified in the schistosome genome. Computer predictions of protein structure indicated substantial similarity of the schistosome proteins and the mammalian CD59 family of proteins, which due to their three-finger-shaped spatial conformation are members of the Three-Finger Protein Domain fold superfamily (TFPD). Members of this family of schistosome proteins were also shown to be expressed predominantly during the mammalian stages when worms are exposed to complement and found to be present at the host-interactive surface of schistosomes. Three different methods were employed to test the possible involvement of these proteins in complement inhibition. Our results strongly suggest that these proteins are not involved in the inhibition of complement and that further studies are needed to establish their functional role(s) in schistosomes.

## Introduction

Schistosomiasis is an important parasitic disease, caused by trematode worms of the genus *Schistosoma*, affecting more than 200 million people worldwide, with a further 650 million individuals living at risk of infection, remaining a major public health problem in many developing countries [1].

Adult worms are able to survive for decades in the hostile blood environment of their vertebrate host, apparently unharmed by circulating leukocytes, antibodies and the complement system. Thus, the parasite must have developed strategies to evade the host's immune defenses. One of the most important modifications is the new tegument surface organization that develops immediately after penetration of cercariae into the skin and their transformation into schistosomula. The tegument is a thin syncytial layer that covers the whole parasite, limited by a basal membrane and a multilaminate surface membrane complex, which constitutes the major host–parasite interface [2].

Schistosomula are at first sensitive to complement killing, but rapidly become highly resistant to complement attack by both the Classical [3], [4] and Alternative Pathways [5], [6]. The later developmental stages of the parasite, i.e., the lung schistosomula and adult worms, have also been shown to be refractory to both pathways of complement [3], [6], [7], [8]. Intravascular parasites must also be resistant to a third complement activation pathway - the Lectin Pathway [9]. However, the precise mechanisms of complement resistance have not been fully characterized.

The strategies used by the parasite to subvert complement attack are most likely related to the function and composition of the tegument (reviewed by [10]). The apical membrane is considered poorly immunogenic due to the limited number of exposed proteins and the acquisition of a variety of host molecules, which may mask important surface proteins [11], [12], [13]. Shedding of the outer coat has been considered as a mechanism to eliminate surface-bound complement and immune-complexes [14]. There are also reports of serine proteases in the surface of freshly transformed schistosomula, lung stage and adult worms, that may play a role in complement resistance by cleaving immuno-complexes and complement proteins [15], [16].

One of the most intriguing strategies that has been proposed as a mechanism by which schistosomes escape complement attack involves the presence in the tegument of several complement regulatory proteins, including two of host origin (reviewed by [17], [18]). The binding activity to the complement components C1 [19], [20], C3 [21], [22], [23] and C8/C9 [24], have been reported to be present at the surface of schistosome parasites. It is important to note that while some of these parasite proteins (e.g. paramyosin) have been isolated and shown to interact with complement components *in vitro*, the definitive protective role of these proteins *in vivo* has not yet been demonstrated.

In the last seven years, several proteomics studies attempted to define the tegument protein composition, as well as its surface exposed molecules [25]. From these studies, one surprising finding was the detection of the host complement components C3 and C4, but not those required for formation of the Membrane Attack Complex (MAC), i.e. C5b to C9 molecules, as revealed by biotinylation studies of the tegument surface [12]. A reasonable explanation is that the complement fixation is initiated, but then inhibited to prevent MAC formation. A mouse C3 complement regulatory (Crry)-like molecule has also been detected on the tegument surface by proteomics [12].

Host cells are protected from MAC-mediated lysis mainly by CD59, a 18–21 kDa glycosylphosphatidyl-inositol-linked membrane glycoprotein that inhibits polymerization of C9 by binding to C8α and C9 [26], thus preventing the formation of the cytolytic MAC. Earlier studies indicated that the exposed form of the schistosome muscle protein paramyosin, was able to inhibit the assembly of C5b-9 by binding to C8 and C9; additionally, this protein was reportedly recognized by rabbit anti-human CD59 antiserum [27]. However, the *in vivo* significance of paramyosin-complement interactions still awaits further clarification.

Recently, Wilson and Coulson [28] identified in the schistosome genome six homologues of human CD59, containing 20–30% amino acid identity which rise to >40% if conservative amino acid substitutions are included. One of these molecules (CD59b, formerly Dif 5) was described by our group as a vaccine candidate, due to its up-regulated expression in the schistosomulum stage [29]. Furthermore, in another approach to select vaccine candidates, two members of this family (CD59a and CD59b) were identified within a group of molecules exposed on the parasite's tegument by proteomics and molecular shaving with phosphatidylinositol-specific phospholipase C (PiPL-C) treatment of live adult worms [13]. More recently, two other isoforms similar to CD59 (Smp_166340 and Smp_081920, GeneDB, (http://www.genedb.org/Homepage/Smansoni) were reported as membrane-associated tegumental proteins by proteomic analysis [25]. Therefore, it is tempting to speculate whether these six homologues could act as CD59-like complement inhibitors in schistosomes as part of an immune evasion strategy, especially because two of them were found on the tegument surface.

The CD59 family possesses the Three-Finger Protein Domain fold (TFPD) [30], that is also a feature of proteins with several distinct sequence and structural attributes, such as the receptors of activins, bone morphogenetic proteins, Mullerian inhibiting substance, transforming growth factor-β receptor II, C4.4a (a structural homologue of the urokinase receptor), urokinase/plasminogen activatory receptor (uPAR) and several members of Ly6 family. The Ly6 molecules (lymphocyte differentiation antigens) were among the first cell surface molecules identified in mouse [31] and there is emerging evidence showing their role in cell signaling, cell adhesion and cellular activation [32]. The TFPD superfamily is characterized by the structural conservation of at least six half-cystines forming three disulfide bridges (B1, B2 and B4), five β-strands and one asparagine adjacent to the N-terminal of the last half-cystine from the last disulfide bridge B4 [33]. A very striking characteristic of this domain is the finger-shaped spatial conformation that the amino acid backbone acquires between the two half-cystines of the same disulfide bridge [30].

In the current work, we describe the molecular characterization of seven putative SmCD59-like genes from genome assembly version 5 (http://www.genedb.org/Homepage/Smansoni) and attempt to address their putative biological function. Our data confirms up-regulation in the transition to intra-host stages. However, the functional studies performed with the two CD59-like members (CD59a and CD59b, named in this study as SmCD59.1 and SmCD59.2), previously identified at the host-parasite interface, did not show any complement inhibition activity. Therefore, the function of these seven proteins in schistosomes remains to be established.

## Materials and Methods

### Parasite material

The life cycle of *S. mansoni* (BH strain) was maintained in the laboratory by routine passage through mice and the intermediate snail host *Biomphalaria glabrata. S. mansoni* eggs were extracted from infected mouse livers and miracidia were hatched from *S. mansoni* eggs, as previously described [34]. Schistosomula were cultivated in culture medium after transformation of cercariae, as previously described [35]. Adult worms were obtained by perfusion of the portal hepatic and intestinal veins from hamsters, 7–8 weeks after infection with approximately 100 cercariae.

### Ethics statement

The procedures involving animals were carried out in accordance with the Brazilian legislation (11790/2008). All animals were handled in strict accordance with good animal practice and protocols were previously approved by the Ethical Committee for Animal Research of Butantan Institute (CEUAIB, São Paulo, Brazil), under the license number 603/09.

### Molecular characterization and 3D modeling

The SmCD59 nucleotide sequences were identified searching the v5.0 of *S. mansoni* genome assembly (GeneDB) (http://www.genedb.org/genedb/smansoni/). The search for conserved domains was performed using SMART (http://smart.embl-heidelberg.de/). The molecular weight (MW) and isoelectric point (pI) were calculated with the Compute pI/Mw tool (http://www.expasy.ch/tools/pi_tool.html).

Post-translational modification predictions: the signal peptide prediction was performed using the SignalP 4.0 server (http://www.cbs.dtu.dk/services/SignalP/) [36]; potential GPI-modification sites were analyzed by big-PI Predictor (http://mendel.imp.ac.at/sat/gpi/gpi_server.html) [37]; and N-glycosylation sites with the NetNGlyc version 1.0 algorithm. Protein sequences alignments were performed using the ClustalX 2 software. Homology modeling was done with MODELLER [38] using the crystal structure of human CD59 (hCD59) (2UWR) as a template. The modeled structure was visualized with PyMOL (The PyMOL Molecular Graphics System, Version 1.5.0.4 Schrödinger, LLC) and the stereochemical quality of the model was examined using the program PROCHECK [39], which evaluates the geometry of residues in the model when compared with the stereochemical parameters from the template. Additional algorithms such as WHAT_CHECK, ERRAT, VERIFY_ 3D, PROVE and CRYST1 record matches (available from: http://services.mbi.ucla.edu/SAVES/) were also used to assess the quality of the model generated.

### Sequence and phylogenetic analysis

We searched the *Schistosoma* Genomic Resources SchistoDB (V3.0) (http://schistodb.net/schisto/) and the GenBank (http://www.ncbi.nlm.nih.gov/) to identify similar sequences in *S. mansoni*, *S. japonicum* and *S. hematobium*, using SmCD59.1 and SmCD59.2 as queries. Additionally, BLAST and PSI-BLAST searches against the non-redundant protein sequence database were used to identify similar sequences in other Platyhelminthes (http://smedgd.neuro.utah.edu/blast.php and http://bioinfosecond.vet.unimelb.edu.au), as well as in representative mammals. For phylogenetic analysis, alignments of protein sequences were performed using the ClustalX 2 software. The tree was constructed using ClustalX 2 using the Neighbor-Joining method. The numbers represent the confidence of the branches assigned by bootstrap (in 1000 samplings). The TreeView program [40] was used to visualize and analyze the tree.

### Real-time RT-PCR

In order to establish the level of expression of each SmCD59 gene throughout the parasite life cycle, total RNA was extracted from adult worms using TRIzol (Life Technologies) and from schistosomula, cercariae, miracidia and eggs using the Kit RNAspin mini (GE Healthcare, USA), as per the manufacturer's recommendations. The RNA was quantified by spectrophotometry (NanoDrop 1000, Thermo Fischer Scientific) and the quality was analyzed in the Agilent 2100 Bioanalyzer. The cDNA synthesis and the quantitative Real-Time PCR (qRT-PCR) reactions using SYBR Green (Life Technologies) were performed according to [41]. The primers were designed in the software Primer Express (Applied Biosystems) to span exon/exon boundaries avoiding amplification of contaminating genomic DNA (Table S1). *S. mansoni* alpha-tubulin (Smp_090120.1) was chosen as normalizing gene. Quantitation of relative differences in expression between the stages was calculated by the comparative 2^−ΔΔCt^ method [42], using the parasite stage with lowest gene expression as calibrator for each gene independently.

In an attempt to compare the levels of gene expression among the seven different SmCD59 isoforms, we performed qRT-PCR using TaqMan Gene Expression Assay (Life Technologies/Applied Biosystems, CA) as previously described [43]. The life cycle stages examined were cercariae, schistosomula cultured for 11 days and adults. RNA was extracted from each life cycle stage using the Trizol method (Life Technologies, CA) and the cDNA was synthesized using 1 µg of high quality total RNA, pre-treated with TurboDNAse (Life Technologies), oligo(dT) and Superscript reverse transcriptase III (Life Technologies). qRT-PCR was performed using cDNA equivalent to 50 ng total RNA. The set of primers and MGB reporter probe, labeled with 6-carboxyfluorescein (FAM) specific for the detection of each SmCD59 were custom synthesized by Applied Biosystems (Life Technologies) and are shown in Table S1. Primers/probe positions were designed to span exon/exon boundaries to minimize detection of any contaminating genomic DNA. The qRT-PCR reactions were run in triplicate and underwent 45 amplification cycles on the StepOne Plus System Instrument (Applied Biosystems). The 2^−ΔΔCt^ method was employed for relative quantification [42] with *S. mansoni* triose phosphate isomerase (SmTPI, Smp_003990) as the normalizing gene. We used the expression of SmCD59.6 from adult worm stage as calibrator to calculate the relative expression of all other SmCD59 analyzed, because this gene had the lowest expression in adult worms.

### DNA constructs - gene optimization and synthesis

The sequence from *S. mansoni* EST assembled contig SmCD59.2 (Smp_105220, GeneDB) was redesigned excluding the signal peptide sequences and manufactured by DNA 2.0, Inc. USA (https://www.dna20.com/) using DNA2.0 optimization algorithms for expression in *Pichia pastoris* (Table S1). The fragments corresponding to the mature protein sequences for SmCD59.1 (Smp_019350, GeneDB) (from H28 to F126) and SmCD59.2 (from K21 to A97) were digested with *Eco*RI and *Xba*I to generate inserts with overhang ends that were purified and cloned into the same sites for the expression vector pPICZαA (Life Technologies), to produce a protein that contained a C-terminal hexa-histidine tag. The resulting constructs were sequenced to confirm their identity.

The SmCD59.2 was also expressed and purified from *E. coli*. The 5′ and 3′ oligonucleotides were designed using the *S. mansoni* genome assembly sequence (Smp_105220). The SuperScriptTM First-Strand Synthesis System for RT-PCR (Life Technologies) was used to amplify a fragment from C27 to H101 of the mature SmCD59.2 protein. The PCR fragments were purified from agarose gel electrophoresis and digested with *Xho*I and *Kpn*I to be cloned into pAE-6His vector [44] and sequenced to confirm identity.

### Expression and purification of recombinant SmCD59.1 and SmCD59.2 in *Pichia pastoris*

The plasmids containing the gene fragments pPICZ-α-SmCD59.1 and pPICZ-α-SmCD59.2 (optimized sequence), were linearized with *Sac*I and used to transform *P. pastoris* strain GS115 (Life Technologies) by electroporation. Putative multi-copy recombinants were selected following the instructions of the manufacturer. To verify production of the relevant proteins, initial studies were done in small-scale expression conditions, followed by Western blot with anti-His-tag antibody (GE). Fermentation conditions for selected clones were carried out in BMGY media (15 mL) at 28–30°C in a shaking incubator until cultures reached an OD_600_ = 2.0 (approximately 16–18 h), as per manufacturer's recommendations. Induction was performed by addition of methanol to a final concentration of 0.5% every 24 h; expression was monitored at 48 and 96 h time points. The supernatants and cell pellets for 10 colonies of each SmCD59 were analyzed for protein expression by Western blot. The colonies that presented the highest expression level were selected for scale-up fermentation.

For protein expression and purification, selected clones were scaled-up for growth in 300 mL in 2.0 L baffled flasks under the same conditions. Cells were harvested after 96 h by centrifugation. The culture medium containing the secreted proteins were filtered through a 0.22 µm membrane, and diluted with 3 volumes of equilibration buffer (20 mM sodium phosphate, 300 mM NaCl, 10 mM imidazole, pH 7.4 (for rSmCD59.1) and pH 8.0 (for rSmCD59.2). The recombinant proteins were then purified by metal affinity chromatography using the ÄKTAprime system (GE Healthcare) under native conditions. Briefly, the sample was loaded onto a Ni^2+^-NTA column (5 mL bed volume) pre-equilibrated with the same buffer. The column was washed with 20 bed volumes of the equilibration buffer and then eluted with a 20–500 mM imidazole linear gradient. Fractions encompassing the main peak were characterized by sodium dodecyl sulfate polyacrylamide gel electrophoresis (SDS-PAGE). Eluted fractions containing the recombinant protein in near pure form were pooled and submitted to extensive dialysis in Phosphate Buffer Saline pH 7.4 (PBS). This sample was analyzed by SDS-PAGE and stained with Schiff's reagent (Sigma) for detection of glycoproteins as per the manufacturer's recommendations. Bovine Serum Albumin (BSA) (Bio-Rad) (non-glycosylated protein) and rSmVAL4 (glycosylated protein) were used as controls for the specificity of the reaction [45]. The rSmCD59.1 and rSmCD59.2 proteins expressed in *P. pastoris* were glycosylated (products between 14.4 kDa and 20.1 kDa), based on their staining with Schiff's reagent and increased size in SDS-PAGE (as can be observed by the protein band shift in comparison to the rSmCD59.2 expressed in *E. coli* (Figure S1). Both recombinant proteins were used in the hemolytic assay and to generate polyclonal antibodies in rats.

### Expression of recombinant SmCD59.2 in *Escherichia coli*

The pAE-SmCD59.2 was transformed into *E.coli* BL21 (SI) (Life Technologies) and the transformed cells were grown in 300 mL LB ON plus ampicillin (100 µg/mL) until they reached an OD600 = 0.7, after which induction was performed by addition of 300 mM sodium chloride (NaCl) for another 4 h at 30°C. Harvested cells resuspended in 30 mL of lysis buffer (20 mM Tris pH 8.8, 150 mM NaCl) were lysed in a French Press. The pelleted inclusion bodies obtained by centrifugation at 20,000× *g* for 30 min were washed twice with wash buffer (lysis buffer, 2% Triton X-100, 2 M urea), and finally resuspended in solubilization buffer (lysis buffer, 10 mM imidazole, 8 M urea). The recombinant protein was refolded from the inclusion bodies by diluting 200-fold into equilibration buffer (solubilization buffer without urea). The recombinant protein was then purified by metal-affinity chromatography using the ÄKTAprime system under native conditions. Briefly, the sample was loaded onto a Ni^2+^-NTA column pre-equilibrated with equilibration buffer. The column was washed with 10 bed volumes of the equilibration buffer and then eluted with 10–500 mM imidazole linear gradient. The main peak was pooled and the protein purity of fractions was assessed by SDS-PAGE. Before its use the protein was dialyzed against PBS, pH 7.4. This sample was used in the hemolytic assay and to generate polyclonal antibodies in rats.

Polyclonal rat antiserum was produced against the preparations of rSmCD59.1 (*P. pastoris*) and rSmCD59.2 (*E. coli*). Rodents were inoculated three times subcutaneously, at 15-day intervals with 100 µg of protein mixed with TiterMax adjuvant (CytRx Corporation; first dose) or PBS (in subsequent doses). Fifteen days after the last inoculation, rodents were exsanguinated.

### Schistosome protein extraction

Total protein extracts from eggs, miracidia, cercariae, 7 day-old schistosomula and adult worms of *S. mansoni* were prepared as previously described [41]. The tegument extract was obtained using a freeze/thaw/vortex procedure [46].

Tegument surface membranes (Tsm) and tegument-extract without-surface membranes (Twm) were obtained after a low speed centrifugation (100× *g*, 30 min) (adapted from [46]). Additionally, soluble (Sol) and insoluble (Ins) fractions of stripped worms after tegument removal were prepared as previously described [47]. The protein extract concentrations were determined with a RC DC Protein Assay Kit (Bio-Rad, CA, USA). Purified rSmCD59.1 or rSmCD59.2 (100 ng) and different parasite extracts (20 µg) were subjected to SDS-PAGE. The gel was electroblotted onto PVDF membrane, which was blocked with 0.02 M Tris (pH 7.5) and 0.3% Tween 20 containing 5% dry milk for 16 h at 4°C. The membranes were incubated in 1∶2,000 or 1∶5,000 dilution of anti-rSmCD59.1 and anti-rCD59.2 primary antibody, respectively in blocking buffer plus 150 mM NaCl for 3 h at room temperature. After three washes using 150 mL of 10 mM Tris (pH 7.5), the membranes were incubated in a 1∶5,000 dilution with secondary goat anti-rat IgG conjugated to horseradish peroxidase (Sigma) for 1 h, followed by another three washes using the same buffer. Antibody reactivity was developed with ECL reagent (GE Healthcare) according to the manufacturer's instructions and imaged using Hyperfilm or Image Quant LAS (GE Healthcare).

### Indirect immunofluorescence and confocal microscopy

Immunocytochemistry on whole adult worms followed a previously described protocol [48]. Briefly, adult worms were fixed in 4% paraformaldehyde for 4 h, washed in PBS (0.1 M, pH 7.4) for 1 h and then transferred to a fresh fixative for another 3 h. After permeabilization with 1% Triton X-100, 0.1% SDS, 10% (heat-inactivated) rabbit serum, 0.1% NaN_3_ in PBS overnight at 4°C, the worms were incubated with primary antibody, diluted 1∶200, for 96 h at 4°C. After extensive washes, the worms were incubated for 48 h with 100 ng/mL of Phalloidin-rhodamine (Molecular Probes, Life Technologies), to stain the musculature of the parasite, and with Alexa Fluor 488-labeled rabbit-anti-rat antibody (1∶200, Molecular Probes, USA) in PBS containing 0.1% Triton X-100, 1% BSA, 0.1% NaN_3_ and 10% rabbit serum at 4°C. After several rinses, the worms were visualized with a LSM 510 Meta confocal microscope (Zeiss), attached to a Zeiss Axiovert 100 microscope.

For cryosection analysis, perfused adult worms were embedded in OCT medium (Tissue-Tek, Sakura) in a pre-cooled beaker of isopentene, frozen in liquid N_2_. Eight-micrometer cryostat adult worm sections were obtained and adhered to silanized glass slides (DakoCytomation, USA) and fixed in acetone for 30 min at −20°C before blocking with 1× PBS, 10% Naive rabbit serum and 0.1% Tween 20 (PNT) overnight at 4°C. They were then incubated with anti-rSmCD59.1 and anti-rSmCD59.2 anti-serum diluted 1∶100 in PNT for 4 h at room temperature. After five washes with PBS 0.1% Tween 20, pH 7.4 (PBS-T), an Alexa Fluor 488 conjugated anti-rat IgG (1∶200) (Life Technologies) and 20 µM DAPI (4′, 6-diamidino-2-phenylindole dihydrochloride, Molecular Probes) to visualize nuclei were added to the PNT solution and samples were incubated for 1 h at room temperature. Sections were washed five times, and then mounted in Fluorescent Mounting Medium (DakoCytomation). Rat pre-immune sera were used as negative control. Images were acquired as described above.

### Hemolytic assays

Briefly, to evaluate Alternative Pathway activity, washed rabbit erythrocytes (ERs) were diluted in AP-CFTD (144 mM NaCl, 0.96 mM sodium barbital, 2.48 mM barbituric acid, 1.4 mM MgCl_2_, 10 mM EGTA) and then added to Normal Human Serum (NHS) (serial dilutions 1∶4 to 1∶256). After incubation at 37°C for 30 min, VBS^−^EDTA (144 mM NaCl, 0.96 mM sodium barbital, 2.48 mM barbituric acid, 20 mM EDTA) was added to stop lysis. After centrifugation (0.8× *g* for 10 min at 4°C) the supernatant absorbance was measured at 405 nm and percentage hemolysis was calculated using ERs lysed by water as the 100% reference. The serum volume that produced 50% lysis of ERs was determined and used in the inhibition assays [49], [50]. To evaluate the hemolytic activity mediated by the Classical Pathway, washed antibody-sensitized sheep erythrocytes (EAs) were diluted in VBS^++^ (144 mM NaCl, 0.96 mM sodium barbital, 2.48 mM barbituric acid, 0.83 mM MgCl_2_, 0.25 mM CaCl_2_) and were added to NHS (serial dilutions 1∶20 to 1∶500). After incubation at 37°C for 30 min, samples were centrifuged (0.8× *g* for 10 min at 4°C) and the supernatant absorbance at 405 nm was measured. The hemolysis percentage (relative to EAs suspension completely lysed by water) was calculated. The volume of serum necessary to promote 50% lysis of EAs was determined and used in the inhibition assays [50].

Inhibition assays were performed to evaluate if the proteins rSmCD59.1 and rSmCD59.2 (produced in *P. pastoris* and in *E. coli*) were able to protect ERs from the lysis triggered by the Alternative Pathway, or EAs from the lysis by the Classical Complement Pathway. BSA (Sigma-Aldrich) was used as a negative control. Different amounts of the proteins (0, 2, 5 and 10 µg for the Alternative Pathway, and 0, 1, 2 and 4 µg for the Classical Pathway) were pre-incubated with NHS (corresponding to 50% lysis) for 20 min at 37°C. Washed ERs (3.5×10^6^) or EAs (6.7×10^4^) were incubated with treated NHS for 30 min at 37°C. After centrifugation (0.8× *g* for 10 min at 4°C), the supernatant absorbance at 405 nm was measured and % hemolysis was calculated.

### SmCD59.1 and SmCD59.2 transfected CHO cells

CHO cells were obtained from American Type Culture Collection (Manassas, VA) and cultured as monolayers in 100 mm cell culture dishes using DMEM/F12 medium (Life Technologies) supplemented with 10% fetal calf serum (FCS), 2 mM L-glutamine, 100 units/mL penicillin and 50 µg/mL streptomycin sulfate. Cells were maintained at 37°C and 5% CO_2_ and reseeded twice a week using 0.05% trypsin.

The full size SmCD59.1 and SmCD59.2 coding regions, including the putative domains for the signal peptide and GPI anchor, respectively, were codon optimized for human and hamster codon preferences, synthesized (Genscript USA Inc., Piscataway, NJ) (Table S1) and cloned into pcDNA-3.1(+) via *Bam*HI/*Xho*I restriction sites. A full length hCD59 cDNA obtained from Open Biosystems/Thermo Scientific, AL was sub-cloned into pcDNA to serve as a positive control for protein expression and complement inhibitory studies in CHO cells. The recombinant plasmids were transiently introduced into 70–80% confluent CHO cells cultured in 6 well-plates using the polymer-based DNA transfection agent jetPEI (Polyplus, France) according to manufacturer's instructions. Transfected cells were cultured for an additional 48 h before harvesting. CHO cells transfected with empty vector were included as negative control.

CHO cells transiently transfected with SmCD59.1 and SmCD59.2 were tested for membrane protein expression. Cells expressing hCD59, or containing empty pcDNA, were included as positive and negative controls, respectively. For fluorescent microscopy and flow cytometry analysis, live cells were washed twice with PBS without CaCl_2_ and MgCl_2_ and detached from the plate with a non-enzymatic cell dissociation solution (Sigma). Cells were washed once with DMEM/F12 containing 0.2% BSA (DMEM-BSA) by centrifugation at 100*× g* for 5 min and resuspended to 10^6^ cells/mL in the same medium. Cells were incubated for 30 min at room temperature with the respective primary antibodies, i.e. CHO-SmCD59.1 with rat polyclonal serum anti-rSmCD59.1 at 1∶500 dilution; CHO-SmCD59.2 with an anti-rSmCD59.2 at 1∶500 and CHO-hCD59 with a rat monoclonal antibody anti-hCD59 at 1∶2,000. After 2 washes with DMEM-BSA, cells were incubated with a secondary FITC-labeled goat anti-rat IgG (Invitrogen, Life Technologies). Cells were immediately visualized under an inverted fluorescent microscope and positive cells were quantified by flow cytometry after subtracting fluorescent background from cells transfected with empty vector. For analysis by Western blot, detached cells were washed twice with PBS and membrane extract was prepared using the ProteoExtract Native Membrane Protein Extraction Kit (EMD Millipore, Billerica, MA), following manufacturer's instructions. The membrane fractions were incubated with each corresponding primary antibody followed by HRP-labeled goat anti-rat IgG (Invitrogen) and developed using the ECL Western Blot Developing System.

To confirm that SmCD59 is GPI-anchored in transfected CHO cells, we followed previously described methodology for hCD59 [51]. Briefly, transfected cells were removed from culture plate wells, washed in Hank's Balanced Salt Solution (HBSS) and exposed to 1 Unit/mL of PiPL-C (Sigma) in HBSS or kept in buffer only as control. After incubation for 1 h at 37°C, cells were stained with the antibody anti-rSmCD59.1 and the percentage of fluorescent cells was quantified and compared with the control sample not treated with PiPL-C using flow cytometry.

### Complement resistance of CHO cells expressing SmCD59.1 and SmCD59.2

CHO cells expressing SmCD59.1 and SmCD59.2 were tested in a complement mediated cell damage assay to determine whether these proteins have complement regulatory activity. This approach was performed according to previously reported methodologies described for hCD59 [51], [52], with some modifications. Briefly, CHO cells transiently transfected with SmCD59.1, SmCD59.2, hCD59 and empty vector were detached from 6-well culture plates as described in the previous section, washed and incubated with 5% rabbit anti-CHO membrane serum in DMEM-BSA for 30 min at room temperature, washed twice with DMEM-BSA and incubated with 10% NHS (Complement Technology, USA) in DMEM-BSA as source of human complement factors. Following 1 h sample incubation at 37°C, cell viability was determined by adding the vital dye propidium iodide at 5 µg/mL and the population of live cells (not stained by propidium iodide) were identified by flow cytometry. Samples with NHS heated at 56°C for 30 min to inactivate complement were used to measure background cell mortality.

### Suppression of SmCD59.1 and SmCD59.2 gene expression by RNAi

Gene-specific siRNAs were commercially synthesized (Integrated DNA Technologies, Inc., IA) and used to induce gene expression knockdown of SmCD59.1 and SmCD59.2, respectively, by electroporation as described [53]. The DNA sequence for SmCD59.1 siRNA is 5′-CTACAAGTGACTAGTCGTAGTTGTG-3′, spanning coding DNA positions 193–217. SmCD59.2 siRNA sequence is 5′-GGTAAAGCTGGCTTAGTAACTGAAT-3′ spanning coding DNA positions 241–265. The negative control siRNA was acquired from IDT and has been previously tested by our own group [53]. Briefly, freshly transformed schistosomula were placed into electroporation buffer (Bio Rad, CA) containing a mixture of SmCD59.1 and SmCD59.2 siRNAs at 5.6 µM each or control siRNA (11.2 µM) or no siRNA. Parasites were immediately electroporated and then cultured for 5 days in DMEM/F12 medium containing 10% FCS at 37°C, in an atmosphere of 5% CO_2_. Reduction in target transcript levels was measured by qRT-PCR relative to transcript levels in parasites treated with control irrelevant siRNA (100% gene expression). Decrease in SmCD59.1 and SmCD59.2 protein levels was measured in whole parasite lysates by Western blots. Schistosomula lysates were prepared by adding 80 µL of RIPA buffer containing a cocktail of protease inhibitors followed by incubation on ice for 1 h. The protein content in each extract was estimated using the BCA Protein Assay Kit (Pierce, IL) according to the manufacturer's instructions. Soluble protein (800 ng) was subjected to SDS-PAGE, blotted onto PVDF membrane and blocked with PBS containing 0.1% Tween 20 and 5% milk for 1 h at room temperature. The membrane was then probed overnight at 4°C with anti-rSmCD59.1 or anti-rSmCD59.2 immune rat serum at 1∶500 dilution or rabbit antibody directed against the schistosome protein aquaporin (SmAQP) as loading control at 1∶250 dilution. Bound primary antibodies were detected by appropriate secondary antibodies conjugated to horseradish peroxidase and exposure to ECL substrate. The same membrane was probed three times to detect SmCD59.1, SmCD59.2 and the control protein, SmAQP. For each re-use, the membrane was stripped with Western Blot Stripping Buffer (Thermo Scientific, IL) following manufacturer's instructions to remove bound antibody.

### Complement killing assay of SmCD59.1 and SmCD59.2 suppressed schistosomula

SmCD59.1 and SmCD59.2 suppressed schistosomula were tested in a complement killing assay modified from Deng *et al.* [27]. Briefly, 200 control and siRNA-suppressed parasites cultured for 5 days were washed in DMEM/F12 and incubated for 30 min at 37°C with serum (1∶4 dilution) from rats infected twice with cercariae (IRS) or with naive rat serum (NRS), both heat-inactivated. Parasites were washed with medium and incubated overnight at 37°C with undiluted NHS as a source of complement. Suppressed and control schistosomula were also tested for complement killing in the absence of rat serum. After 18–24 h, schistosomula were exposed to 1 µg/mL of DNA binding stain Hoechst 33258 dye for 5 min and examined with an inverted fluorescent microscope to count the number of dead fluorescent parasites under ultraviolet light (352 excitation/455 emission) [54]. Dye uptake only occurs if there is complement induced-tegumental damage. Samples were run in duplicates in three independent experiments. Percent net mortality was calculated by subtracting background mortality of parasites treated with heat-inactivated NHS. The same complement killing assay protocol was used to measure mortality of freshly transformed schistosomula cultured for 3 h in DMEM/F12 plus 10% FCS.

## Results

### Sequence and *in silico* analyses

**Figure 1:**
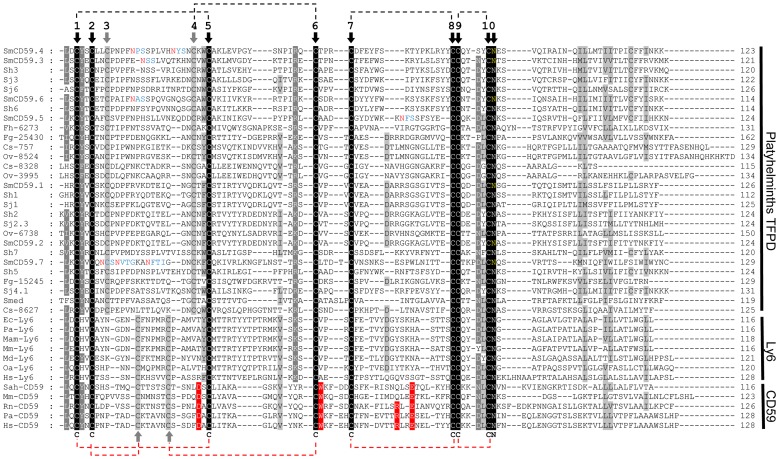
ClustalX multiple sequence alignment of the mature protein sequence (excluding the signal peptide) of TFPDs from platyhelminthes, CD59 and Ly6. The regions with high identity and similarity between sequences are shown as black and gray columns, according to the ClustalX algorithm. Arrows indicate highly conserved Cysteines and Asparagines with a C and an N, respectively. Dashed lines represent pairs of cysteine residues forming disulfide bonds determined from Hs-CD59 (red) and predicted for SmCD59.2 (black). Only for SmCD59 sequences, potential sites for N-glycosylation are shown in blue with asparagine (N) in red, and potential sites for GPI anchor are shown in yellow. Human CD59 active sites are shaded in red. The sequences abbreviation are: *Schistosoma mansoni* (SmCD59.1-7), *Schistosoma japonicum* (Sj1, Sj2.3, Sj3, Sj4.1, Sj6), *Schistosoma hematobium* (Sh1-3 and Sh5-7), *Clonorchis sinensis* (Cs-757, Cs-8328, Cs-8627), *Opisthorchis viverrini* (Ov-8524, Ov-3995 and Ov-6738), *Fasciola hepatica* (Fh-6273), *Fasciola gigantica* (Fg-25430 and Fg-15245), *Schmidtea mediterranea* (Smed), *Equus caballus* (Ec-Ly6), *Pongo abelii* (Pa-Ly6 and Pa-CD59), *Macaca mulatta* (Mam-Ly6), *Mus musculus* (Mm-Ly6 and Mm-CD59), *Monodelphis domestica* (Md-Ly6), *Ornithorhynchus anatinus* (Oa-Ly6), *Homo sapiens* (Hs-Ly6 and Hs-CD59), *Saimiriine herpesvirus* (Sah-CD59), *Rattus norvegicus* (Rn-CD59) (the accession numbers are listed in the Table S2).

BlastP comparisons of SmCD59.1 and SmCD59.2 sequences to GenBank revealed low similarity with orthologs of CD59 proteins belonging to the uPAR/Ly6/CD59/snake toxin-receptor superfamily (E-value, ranging from 3×10^−10^–4×10^−4^). Because this is the first article to deal specifically with this schistosome gene family, herein we propose numbering of these genes as shown on Table 1. In addition to the six previously reported *S. mansoni* sequences, SmCD59.1-6, it was possible to identify a new *S. mansoni* member (SmCD59.7 – Sm_125250) (Table 1), which displays a slightly different distribution of Cys 10 (Figure 1). Furthermore, nine sequences from *S. japonicum* and six from *S. hematobium* could be identified, and all branched together with the *S. mansoni* members representing possible orthologs (Figure S2). In spite of the low bootstrap value, all these sequences did not branch together with mammalian Ly6 or CD59 sequences. Additionally, searching other databases, 13 non-characterized proteins from other Platyhelminthes species were identified (E-value, ranging from 9×10^−19^–2×10^−7^). Noteworthy, was the identification of a member in the turbellarian *S. mediterranea* (Figure S2).

**Table 1:** Molecular characteristics of *Schistosoma mansoni* CD59-like proteins.

Name^a^	*S. mansoni* v5 ID^b^	pI/Mw^c^ (kDa)	Ortholog^d^ Organism (% id)	Primary structure analysis^e^			Location^f^ Expression
				Signal Peptide	Domain	GPI anchor	
SmCD59.1	Smp_019350	8.11/11.0	Ly-6 *Sarcophilus harrisii* (34%)	x	uPAR/Ly6/CD59/snake toxin	x	Tegument^1^ ↑Day 3 Sch^2^
SmCD59.2	Smp_105220	8.33/12.1	Ly-6D *Macaca mulatta* (33%)	x	uPAR/Ly6/CD59/snake toxin	x**	Tegument^1^ ↑Day 3 Sch^2^^, ^ 3
SmCD59.3	Smp_081900.2	8.47/11.4	CD59 *Myotis davidii* (30%)	x		x**	↑ Day 3 Sch^2^
SmCD59.4	Smp_166340	8.65/11.5	Ly-6 *Desmodus rotundus* (34%)	x		x**	Tegument^4^ ↑Day 3 Sch^2^
SmCD59.5	Smp_081920	8.66/11.6	CD59 *Myotis davidii* (29%)	x	uPAR/Ly6/CD59/snake toxin	x**	Tegument^4^ ↑Day 3 Sch^2^
SmCD59.6	Smp_166350	9.00/11.5	CD59 *Canis lupus familiaris* (27%)	x	uPAR/Ly6/CD59/snake toxin	x**	
SmCD59.7	Smp_125250	8.95/11.4	Ly-6D *Saimiri boliviensis* (24%)	x*		x**	

a Proposed gene names (accession numbers of cloned cDNAs).

b Schisto GeneDB version 5 systematic ID.

c Molecular weight and isoelectric point.

d BLASTx analysis for identification of the closest ortholog in GeneBank; ortholog protein, organism, identity.

e SMART predicts the presence of domains in the protein sequence.

f Location of the protein in proteomic studies and up regulated stages by microarray analysis;

(1) Castro-Borges et al., 2011;

(2) Parker-Manuel et al., 2011;

(3) Farias et al., 2010;

(4) Wilson, 2012.

* Signal peptide prediction with SignalP 4.1 server (*D-scores* above 0.4 but below 0.5).

** GPI Prediction Server (Version 3.0) bellows the cut-off.

Analyses of the primary sequences revealed the presence of ten conserved cysteine residues following the same pattern of distribution in Platyhelminthes (Figure 1). A very intrinsic characteristic of the TFPD fold is the presence of at least four conserved cystines among all the different classes of proteins having this domain [30]. Nonetheless, this pattern differs from the CD59s of mammals in the 3^rd^ and 4^th^ cysteines, which are separated by a larger stretch of amino acids in the Platyhelminthes sequences and are located at a distance of exactly two amino acids from their closest cysteines (2^nd^ and 5^th^ Cys). These distances (from the 2^nd^ to the 3^rd^ and from the 4^th^ to the 5^th^ Cys) in mammals are more variable. However, the most important difference of these molecules when compared to human CD59, is that they do not have the conserved amino acid residues (D24, W40, R53 and E56 in hCD59) involved in complement recognition [30], [55], as well as the “hydrophobic groove” (C39, W40 and L54 in hCD59), highly conserved in all CD59s [55]. The sequence belonging to Herpesvirus Saimiri Protein (Sah-CD59) was included as a homolog from human CD59. Similarly, a glycine adjacent to the sixth cysteine (characteristic of toxins) is not present in the sequences [33] (Figure 1).

### Homology modeling

**Figure 2:**
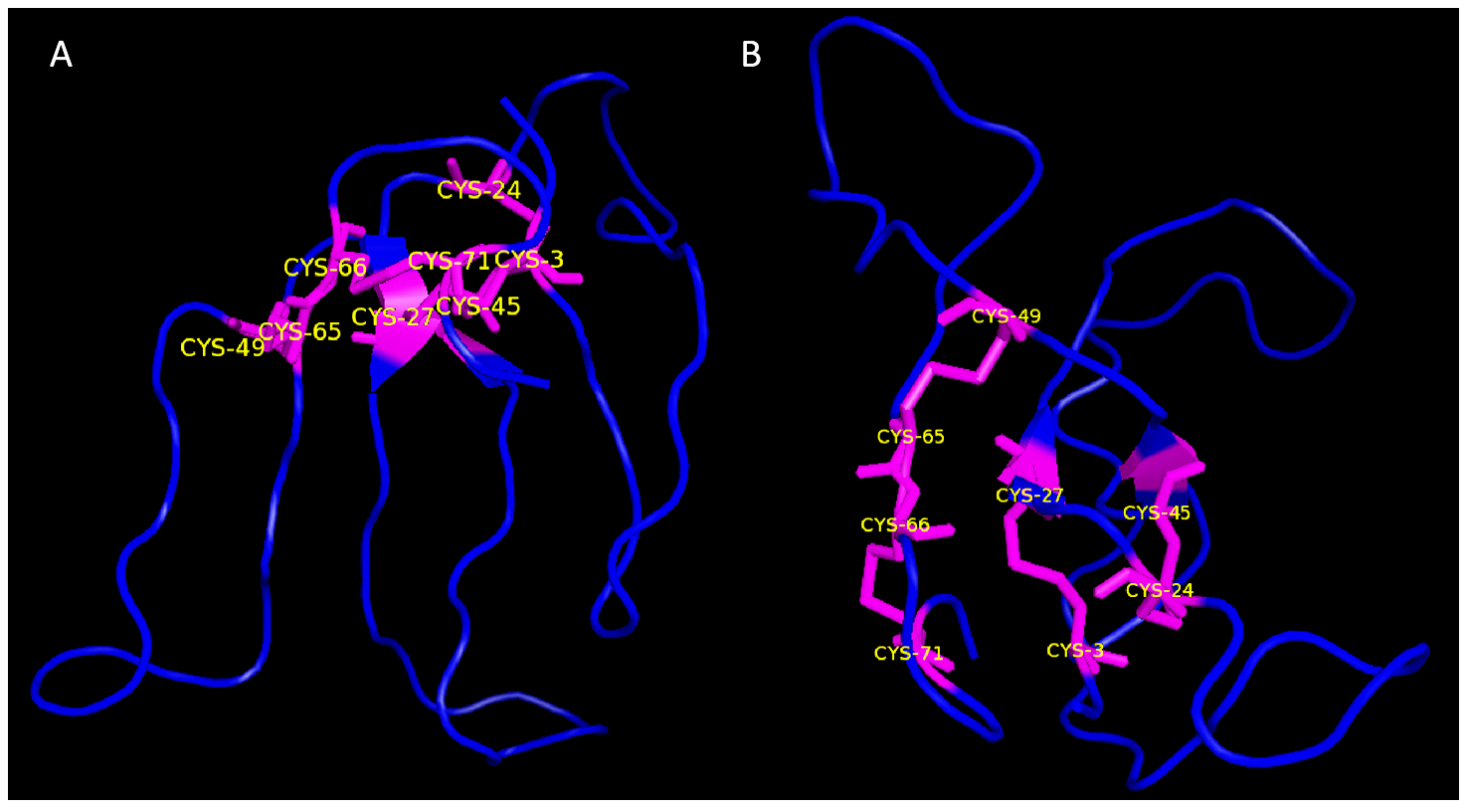
Homology modeling of SmCD59.2. Ribbon display, side view (A) and top view (B). The models were generated with Modeller 5.1 using 3-D structure of human CD59 as a template (2UWR). The quality of the model was assessed with Procheck. Disulfide bridges are visualized as magenta sticks, and the three finger-shaped backbone is visualized as projections emerging from the disulfide bridges.

In order to explicitly visualize the putative domain features and also provide insight into its folding, 3D-modeling of SmCD59.2 (Smp_105220) was performed using human CD59 as a template (2UWR) (Figure 2). Despite the low similarity between the two sequences (∼37%), it was possible to align them in order to perform the modeling. Out of the 124 residues, 73 of them were modeled (beginning at V25, which becomes V1 in the mature protein, and ending at A97, becoming A73). The last 27 residues at the C-terminal remained out of the model, since they did not show any similarity to 2UWR (Figure 1). RMSD was of 1.24 Å between template and models in the common core (36 alpha-carbon atoms).

In this model, the finger-shaped backbone is present within the structure, emerging from a hydrophobic palm, clearly seen in Figure 2A. Additionally, the four conserved cystines can be observed (Figure 2B) and would be formed by the following pairs: Cys3-Cys27, Cys24-Cys45, Cys49-Cys65 and Cys66-Cys71. There are another two conserved cysteines in the *Schistosoma* spp. (Cys6 and Cys9 in SmCD59.2) that may not form a disulfide bridge. However, the possibility of a fifth cystine formed by these two cysteines cannot be ruled out, since the model generated is not necessarily a real portrait of the native molecule's fold. All these spatial conformations are possible since the TFPD signature is present in the sequence (Figure 1). The geometry of the final refined model was evaluated with a Ramachandran plot, which showed that 97% of the amino acid residues were positioned in the “allowed” regions (data not shown).

### Most of the SmCD59 genes are differentially expressed in the transition from cercaria to schistosomulum and adult worms

In order to establish the level of expression in different parasite stages, including cercariae, *in vitro* cultured 7-day old schistosomula, adult worms, eggs and miracidia, qRT-PCR was performed using SYBR Green (Life Technologies). Gene expression fold changes of each SmCD59 were calculated relative to the less expressed stage after normalization to the alpha-tubulin housekeeping gene. The results show that the SmCD59 genes, with the exception of SmCD59.6, display increased gene expression in the schistosomulum and adult worm stages (Figure S3).

**Figure 3:**
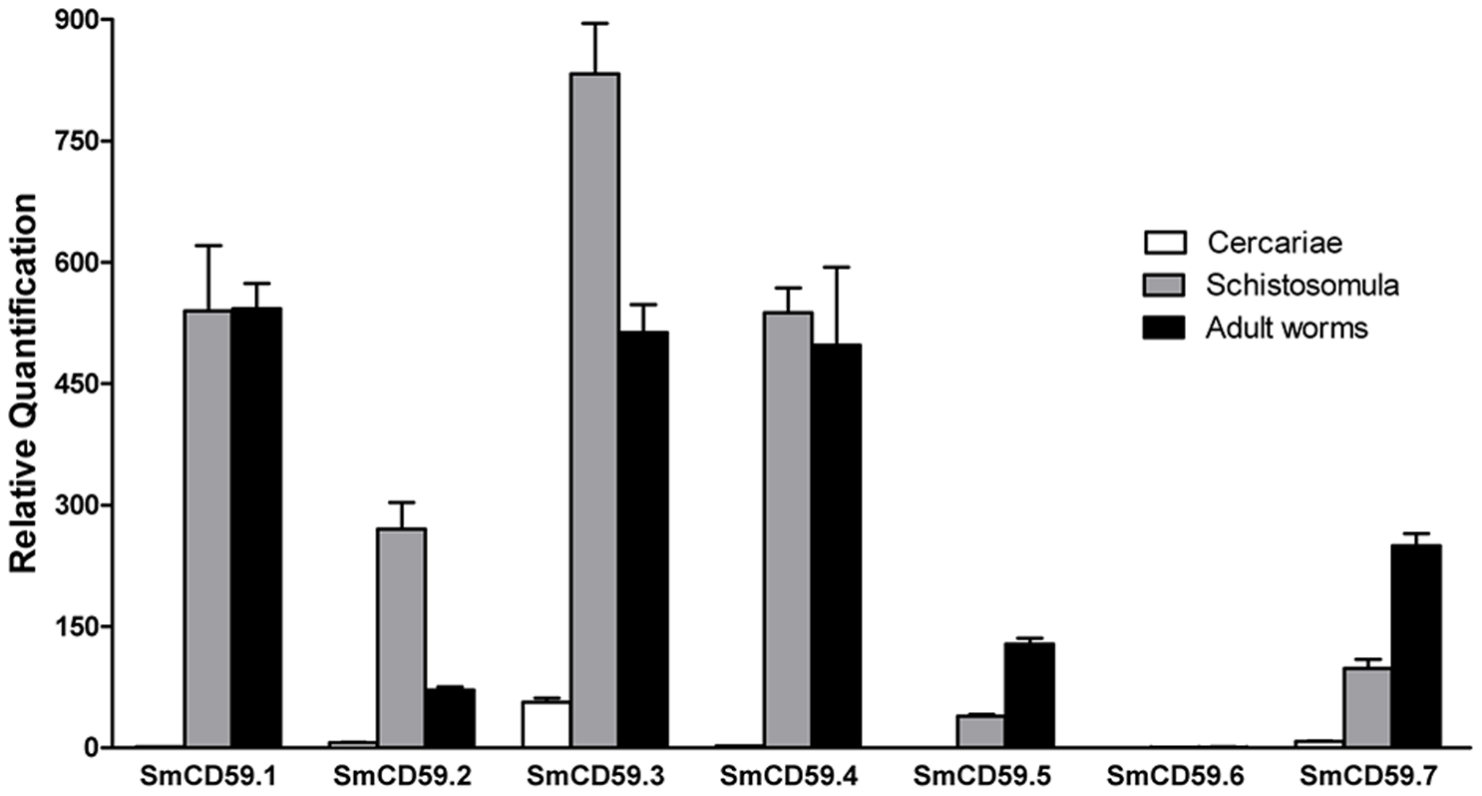
Relative expression levels in different SmCD59 isoforms. Relative gene expression levels for each homologue was determined by qRT-PCR and represented as mean (+ SD) from technical triplicates. The developmental stages examined were cercariae, schistosomula (11-day cultured), and adult pairs (6-week old).

To evaluate the relative expression levels between the different SmCD59 isoforms, qRT-PCR was performed using the Taqman system on three stages, the free living infective stage (cercaria), *in vitro* cultured 11-day old schistosomula and adult worms. SmCD59.1, SmCD59.3 and SmCD59.4 showed the highest levels of expression with higher expression in the schistosomula and adult stages, while SmCD59.2, SmCD59.5 and SmCD59.7 showed intermediate levels of expression and SmCD59.6 was found at very low levels (Figure 3).

### SmCD59 protein expression profile across the parasite life cycle stages

**Figure 4:**
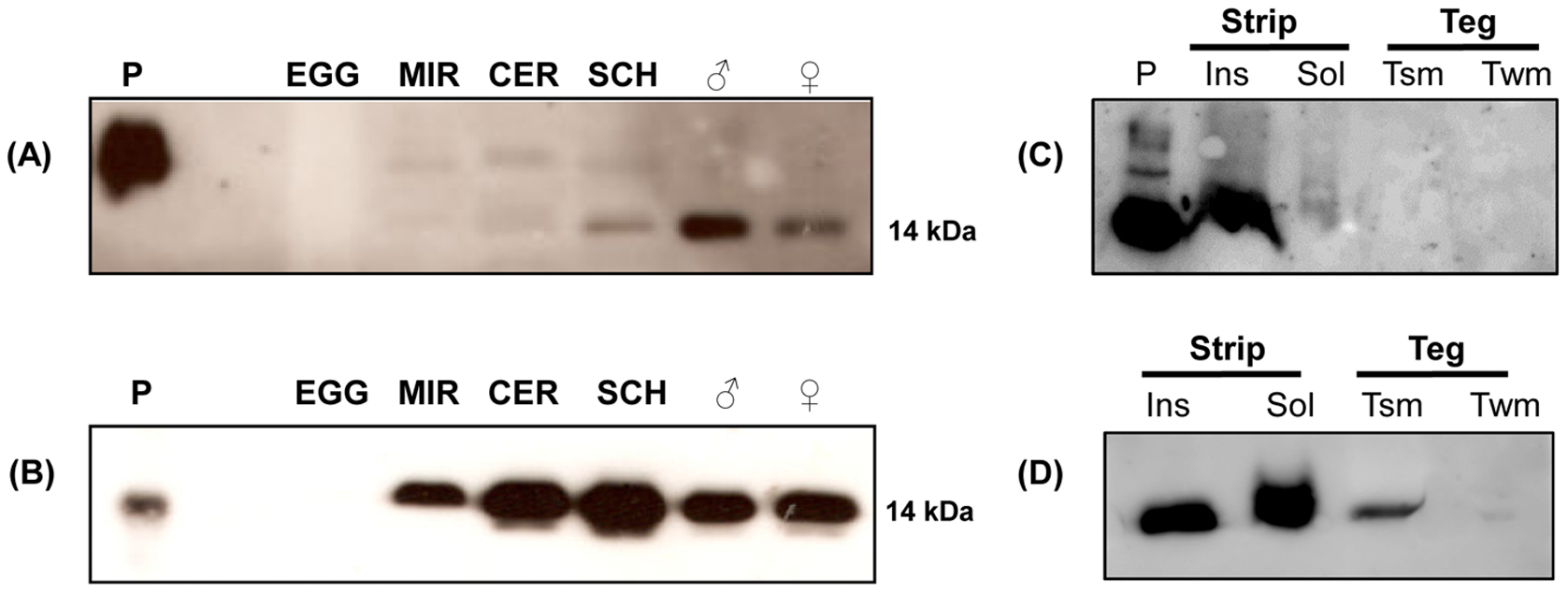
Immunoblotting of protein extracts from *S. mansoni* stages using anti-rSmCD59.1 or anti-rSmCD59.2 polyclonal antibodies. Protein extracts (20 µg) from different *S. mansoni* stages: EGG (eggs), MIR (miracidia), CER (cercariae), SCH (*in vitro* 7-day-old schistosomula), ♂ (male adult worm), ♀ (female adult worm) and P (positive control, 100 ng of rSmCD59.1) were analyzed using (A) anti-rSmCD59.1 antiserum; or (B) anti-rSmCD59.2 antiserum, P (positive control, 100 ng of rSmCD59.2 expressed in *E. coli*). Extracts of stripped worms (Strip) and Tegument (Teg) of adult worms were probed with (C) anti-rSmCD59.1 or (D) anti-rSmCD59.2 antisera. Insoluble (Ins) and soluble (Sol) protein extracts of stripped worms; (Tsm) enriched tegument surface membranes fraction; (Twm) tegument extract without surface membranes. Positions of molecular mass standard are indicated.

Samples prepared from cercariae, schistosomula, adult male and female worms, eggs and miracidia stages from *S. mansoni*, and tegument isolated by the freeze/thaw method, were all separated by 15% SDS-PAGE. Immunoblotting was performed using mouse anti-rSmCD59.1 and anti-rSmCD59.2 antisera. The protein expression profile of SmCD59.1 generally correlated with the Real Time RT-PCR data, revealing low expression in miracidia and cercariae, increasing in schistosomula and adult worms (Figure 4A). A lower intensity band of higher molecular mass may be attributed to some cross reactivity with other SmCD59 members (possibly glycosylated) (Figure 4A), as suggested by the *in silico* predicted N-glycosylation sites (Figure 1). In the case of SmCD59.2, a similar expression profile with higher levels in schistosomula was observed (Figure 4B).

Notably, after the separation of the tegument from the worm body, the SmCD59.1 protein was detected only in the insoluble fraction of stripped worms (Figure 4C). We cannot rule out the presence of this protein in the tegument fraction; however, this data suggested that the protein is much more abundant in denuded worms than in the tegument. The SmCD59.2 protein was found to be more abundant in stripped worms, with similar proportions in the soluble and insoluble fractions (Figure 4D). Furthermore, analysis of the tegument surface membrane fraction revealed that the small amount of protein present in the tegument is membrane associated, with no protein detected in the soluble supernatant (soluble syncytial proteins) (Figure 4D).

### SmCD59.1 and SmCD59.2 present a ubiquitous localization in adult worms of *S. mansoni*

**Figure 5:**
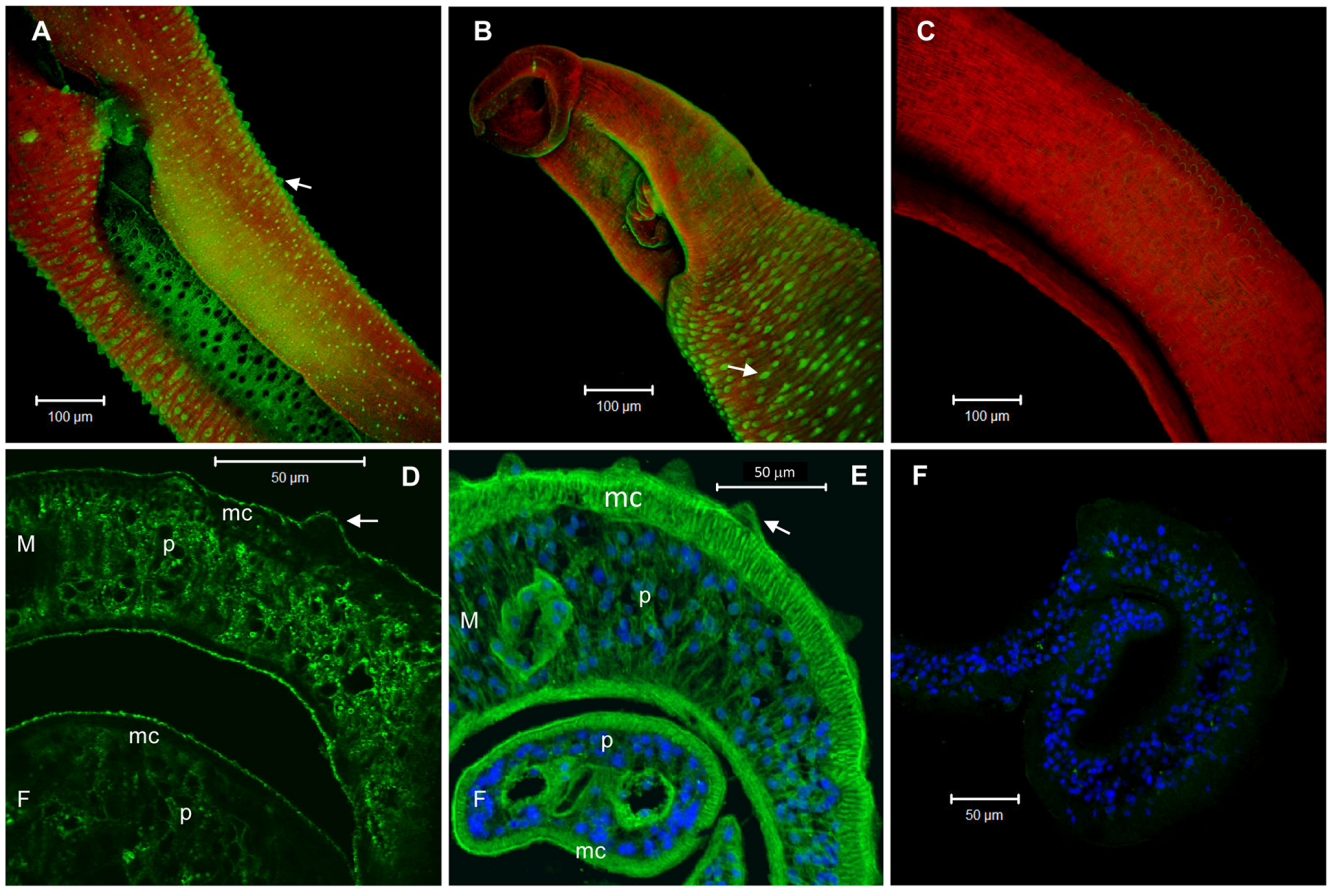
Fluorescence confocal microscopy images showing immunolocalization of SmCD59.1 and SmCD59.2 in whole mount and in transverse sections of *S. mansoni* adult worms. (A and B) Confocal projections of SmCD59.1 and SmCD59.2 protein in the tegument of whole mount adult male worms. (D and E) Fluorescence detection of SmCD59.1 and SmCD59.2 in transverse sections of *S. mansoni* adult worms. (C and F) Negative control, serum from naïve rat. Secondary antibody coupled to Alexa 488 (green) was used for SmCD59 localization. DAPI (blue) was used for nucleus localization (E and F) and Rhodamine Phalloidin (red) was used for actin localization (A, B and C). Arrows – tegument tubercules; M – male; F – female; p – parenchyma; mc – muscle cells.

Immunolocalization studies on whole adult worms using rat serum raised against rSmCD59.1 and rSmCD59.2 revealed that both proteins were expressed on the surface of *S. mansoni* adult male worms, as shown by the green staining apparent around and over their dorsal tubercules (Figure 5A and B). However, analysis of adult worm sections revealed that SmCD59.1 protein is also expressed at significant levels in the parenchyma cells of male and female adult worms (Figure 5D), while SmCD59.2 protein shows even a broader localization, including muscle cells of male and female adult worms (Figure 5E).

### Investigation of the inhibition of complement-mediated hemolysis by rSmCD59.1 and rSmCD59.2 proteins

**Figure 6:**
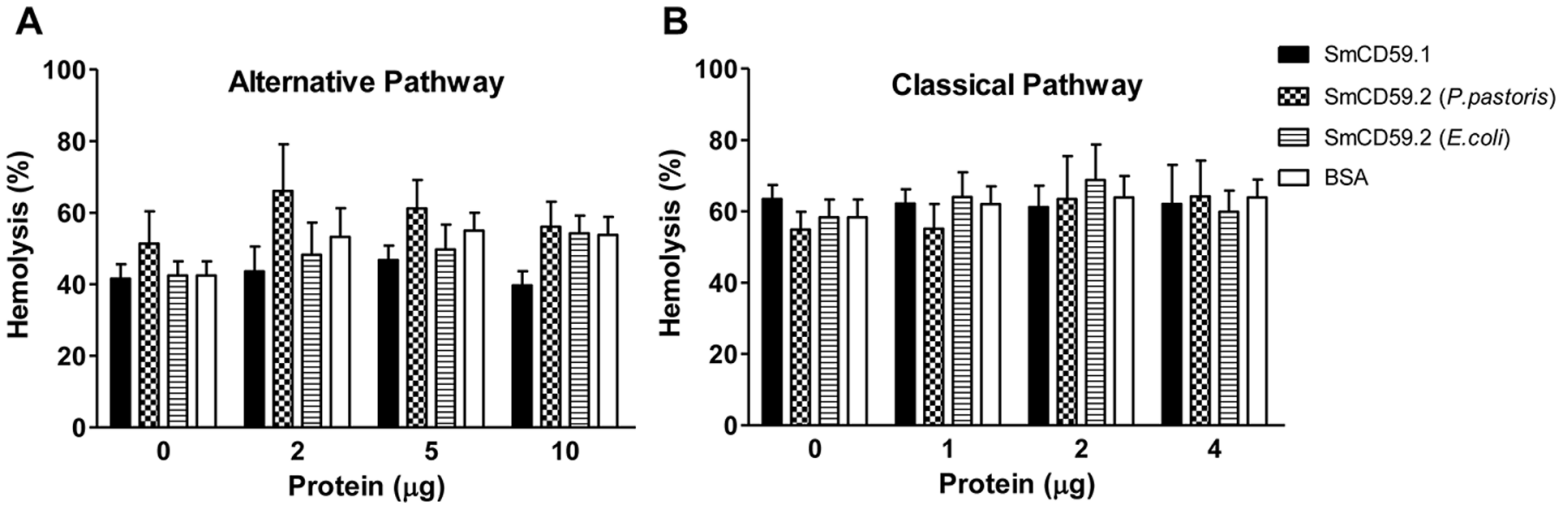
Evaluation of the ability of SmCD59.1 and SmCD59.2 to modulate complement activity. Hemolytic assays were performed after incubating normal human serum (NHS) with different amounts of SmCD59.1 (produced in *P. pastoris*), SmCD59.2 (produced in *P. pastoris* or *E. coli*), or BSA (negative control). (A) Treated NHS was then incubated with rabbit erythrocytes (Alternative Pathway) or (B) antibody-sensitized sheep erythrocytes (Classical Pathway). The percentage of hemolysis was calculated in comparison with erythrocytes suspensions completely lysed with water (100% lysis). The volume of NHS used in these assays corresponds to the amount that promotes 50% lysis of erythrocytes. Each column represents the mean of three independent experiments ± SD.

In order to investigate the involvement of SmCD59 isoforms in the inhibition of the complement cascade, NHS was pre-incubated with various amounts of rSmCD59.1 and rSmCD59.2 proteins, and then added to rabbit erythrocytes (Alternative Pathway) or antibody-sensitized sheep erythrocytes (Classical Pathway). Our data revealed that the rSmCD59 isoforms and the negative control BSA did not inhibit hemolysis triggered by the Alternative or Classical Complement Pathways (Figure 6). The NHS used in these assays promoted 50% erythrocyte lysis and addition of the recombinant schistosome proteins or BSA did not alter this percentage. These results therefore suggest that the rSmCD59 isoforms are not involved in the inhibition of complement deposition by the Classical or Alternative Pathways.

### Evaluation of complement resistance of CHO cells expressing SmCD59.1 and SmCD59.2

It has been shown that the membrane form of hCD59 (GPI-anchored) is about 5 to 10 times more active at inhibiting the complement system as compared to the soluble form lacking the GPI anchor [56], [57]. Thus, CHO cells were transfected with the codon optimized full-length cDNA of SmCD59.1 (CHO-SmCD59.1) and SmCD59.2 (CHO-SmCD59.2) to measure complement resistance of cells expressing these membrane-anchored proteins. Cells transfected with hCD59 (CHO-hCD59) and empty vector (CHO-pcDNA) were included as positive and negative controls, respectively.

Expression of SmCD59.1 in the CHO cell surface was demonstrated by positive staining of the membrane of live cells with an anti-rSmCD59.1 antibody. Flow cytometer analyses confirmed expression of SmCD59.1 in about 20% of the transfected cells (Figure S4A). Similar results were obtained with hCD59 (Figure S4B). Expression of SmCD59.2 in the cell membranes was detected by Western blot of a membrane preparation of transfected cells (anti-rSmCD59.2 rat serum did not react with endogenous CD59 protein in live CHO cells – data not shown). To confirm that SmCD59.1 is GPI-anchored, transfected CHO cells were treated with PiPL-C, labeled with anti- rSmCD59.1 antibody and analyzed by flow cytometry. As shown in Figure S4C (top panels), there was a pronounced reduction in antibody labeling anti-rSmCD59.1 after treatment with PiPL-C (6.8%) as compared to untreated cells (25.8%). Results were similar for CHO-hCD59 cells included as positive control (bottom panels).

**Figure 7:**
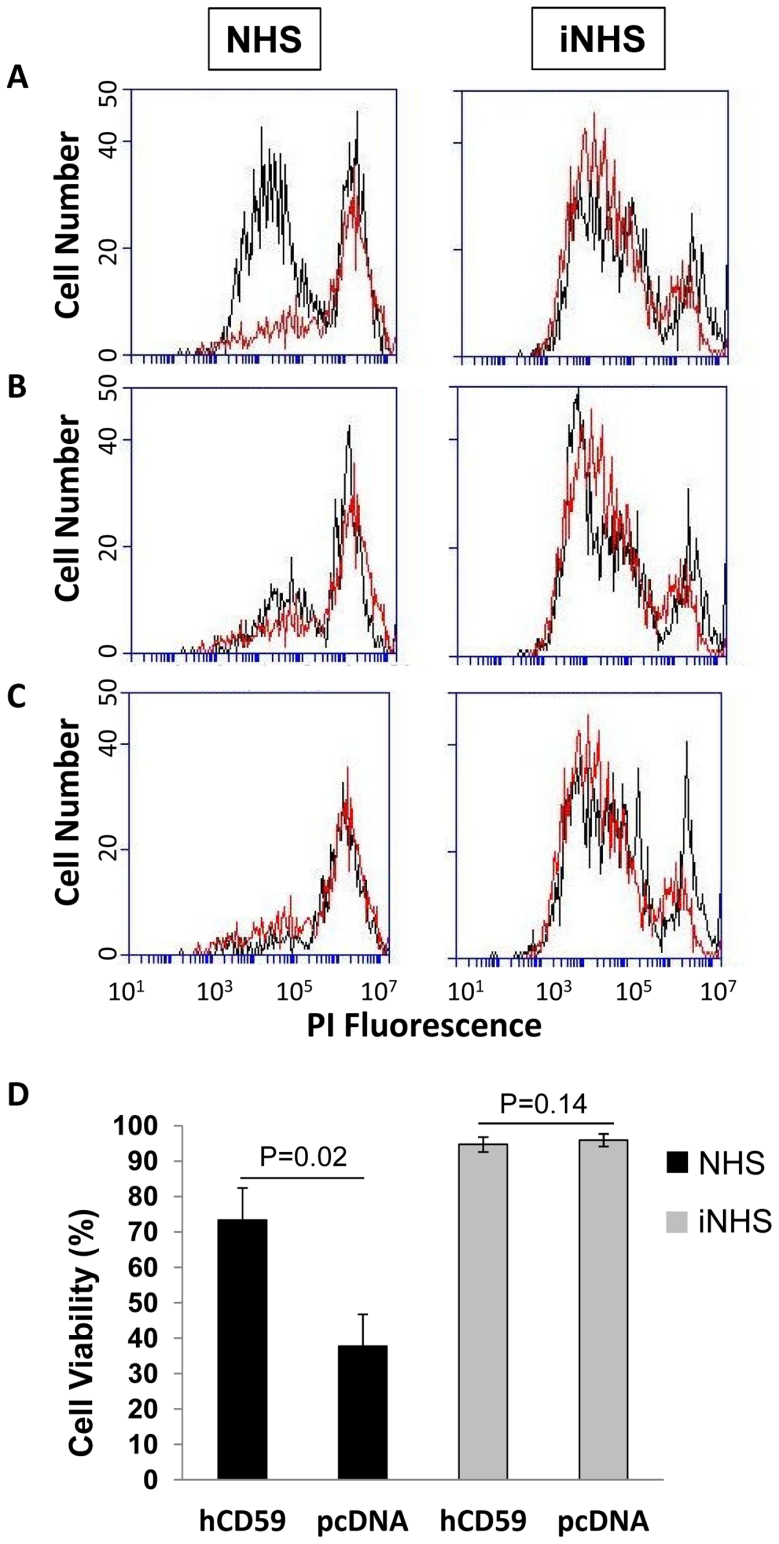
Complement resistance of CHO cells expressing SmCD59.1, SmCD59.2 and hCD59 (positive control). Viable cells were quantified by flow cytometry following exposure to anti-CHO antibodies and normal human serum (NHS) or heat-inactivated serum (iNHS). Black histograms are CHO cells transfected with hCD59 (A), SmCD59.1 (B) or SmCD59.2 (C), respectively. Red histograms are control cells transfected with empty pcDNA vector (A, B and C). The first peak in both black and red histograms represents viable cells and the second peak represents dead cells stained by propidium iodide (PI). (D) Cell viability of CHO cells transfected with hCD59 or pcDNA treated with anti-CHO antibodies and NHS or iNHS in three independent experiments (mean ± SD). P values are indicated.

CHO-SmCD59.1 and CHO-SmCD59.2 were tested for complement resistance when exposed to antibodies reactive to CHO membrane proteins and to complement from NHS. Background cell mortality was monitored by including samples treated with heat-inactivated complement (iNHS). Complement-treated cells were analyzed by flow cytometry to quantify the live cell population, i.e. cells of reduced fluorescence that are not stained by propidium iodide (Figure 7). The number of viable cells in the CHO-hCD59 sample was considerably higher than the number of live cells in the CHO-pcDNA sample after addition of NHS (Figure 7A, first peak in left panel). *T*-test analysis of three independent experiments (Figure 7D) showed that CHO-hCD59 cells were significantly more resistant to complement (73.4%±9.1) than CHO-pcDNA cells (37.8%±9%) (p<0.05). When cells were treated with iNHS, the number of live cells in both samples was nearly identical and close to 95% (Figure 7A, first peak in right panel and Figure 7D), confirming the complement inhibitory activity of hCD59 in the transfected cells. The same cell survival analysis was performed on CHO-SmCD59.1 and CHO-SmCD59.2 samples, but there was no difference in cell viability in both samples compared to CHO-pcDNA sample after exposure to NHS (Figure 7B and 7C respectively). These results strongly indicate that SmCD59.1 and SmCD59.2 are not complement inhibitory proteins.

### Effect of SmCD59.1 and SmCD59.2 gene suppression on complement killing of schistosomula

**Figure 8:**
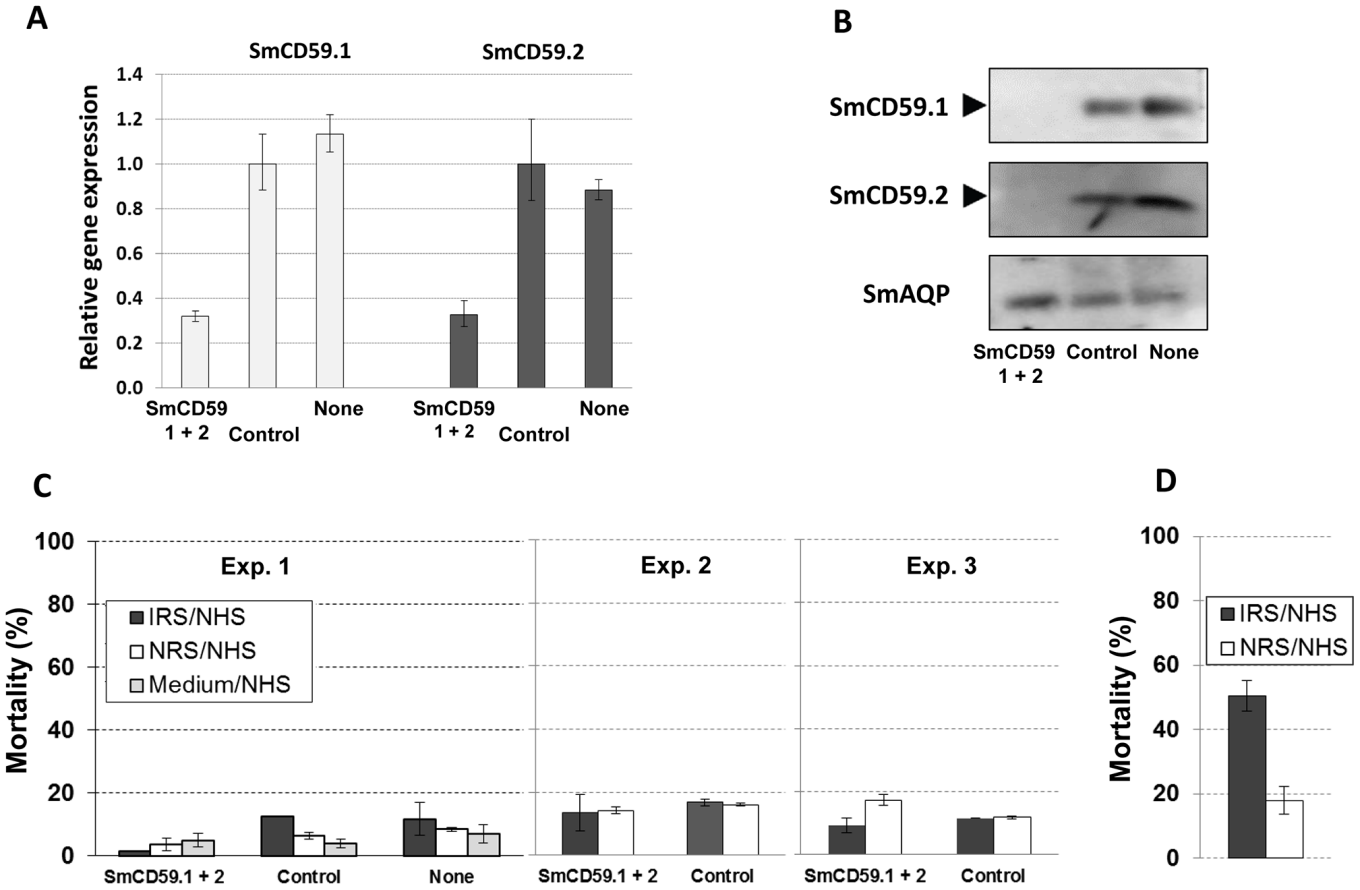
SmCD59.1 and SmCD59.2 gene expression knockdown and complement mediated killing assay of suppressed parasites. (A) Relative SmCD59.1 and SmCD59.2 gene expression (mean ± SE) in schistosomula 5 days after treatment with SmCD59 siRNAs or control siRNA (control) or not treated parasites (none). “SmCD59.1+2” indicates parasites treated with a mixture of equal molar amounts of SmCD59.1 and SmCD59.2 siRNAs. (B) Protein levels in SmCD59-suppressed and control schistosomula groups as described in (A). Western blot results are shown for SmCD59.1 (top panel), SmCD59.2 (middle panel) and the *S. mansoni* aquaporin (SmAQP) as a loading control (bottom panel). (C) The parasites were treated with immune rat serum (IRS), normal rat serum (NRS), or kept in medium only and then treated with normal human serum (NHS) as source of complement. Percent net mortality of schistosomula is shown and was determined by background mortality subtraction of parasites treated with heat-inactivated NHS. Results shown are mean ± SD of duplicate samples in three independent experiments. (D) Freshly transformed schistosomula (3 h in culture) were included as a positive control in the complement mediated killing assay.

To further assess whether SmCD59.1 and SmCD59.2 protect schistosomes from the complement attack, freshly transformed schistosomula were treated with a mixture of SmCD59.1 and SmCD59.2 specific siRNAs and tested for complement susceptibility 5 days later. Transcript levels measured by qRT-PCR were about 60% lower in SmCD59-suppressed parasites than in control parasites (Figure 8A). Schistosomula were suppressed immediately after cercarial transformation because SmCD59 mRNA levels are undetectable by qRT-PCR at that early time point after which expression increases significantly (data not shown). This approach was expected to ensure that SmCD59 gene knockdown would substantially inhibit protein production in siRNA-treated parasites, while control parasites would have abundant protein levels. Indeed, Western blot analysis in Figure 8B confirmed dramatic reduction of SmCD59.1 (top panel) and SmCD59.2 (middle panel) proteins in siRNA-suppressed parasites compared to lysates of control parasites. In the bottom panel, the SmAQP control protein was detected in all lysates, demonstrating that comparable levels of protein were present in each lane. The significant decrease of protein levels in siRNA-suppressed schistosomula did not enhance in vitro parasite killing by human complement beyond 20%, even in the presence of immune serum when compared to the control groups in three independent experiments (Figure 8C). Finally, data in figure 8D was obtained to validate the complement assay and to confirm previous results [3], [4], [7] in which, contrary to older schistosomula, 3 h-cultured parasites are more susceptible to complement killing, particularly when the killing is antibody mediated. Thus, our results indicate that SmCD59.1 and SmCD59.2 are not complement regulatory proteins in schistosomes.

## Discussion

Early analysis of the *S. mansoni* sequences, SmCD59.1 and SmCD59.2, revealed similarity to human CD59 [13], [28], [29]. In the present report, we have described members of the TFPD family in Platyhelminthes and compared their structure and folding to the human CD59 protein [58]. This group of proteins contains the TFPD fold, since the characteristic cysteines of this family are present in their sequences. Despite the fact that the cysteines are conserved, there are two important differences that are evident in the alignment: i) the 3^rd^ and 4^th^ cysteines are in different positions when aligned with their mammalian counterparts; nevertheless, all the Platyhelminthes sequences maintain the two-amino acid distance pattern between the 2^nd^/3^rd^ and 4^th^/5^th^ cysteines; ii) the Platyhelminthes sequences lack the essential amino acids in the active site required to block the formation of the MAC. However, it is reasonable to expect that not all these features would be totally conserved in trematodes and mammals, since these proteins may have hypothetically evolved as complement inhibitors through convergent evolution. For example, there is a fish homologue of CD59, which was shown to have fish complement inhibitory activity, but does not have many of the so-called ‘critical’ residues conserved [59]. Furthermore, the presence of at least one CD59 member in the non-parasitic flatworm, *S. mediterranea*, suggests that these proteins are also participating in aspects of non-parasitic Platyhelminth biology. Moreover, it is at least intriguing how this pattern remained conserved in inferior taxa, while in mammals it seems to be more variable.

The transcription of most SmCD59 genes is up-regulated following the transition from free-living cercaria to the parasitic schistosomula stage; some of these genes continue to show increasing transcription into the adult worm stage. These data confirm previous microarray results showing up-regulation of SmCD59.1-5 expression in 3-day old schistosomula in comparison to germ-balls and cercariae [60]. Since SmCD59.1 and SmCD59.2 are the only representatives of this family confirmed to localize at the host-parasite interface by PiPL-C treatment, they were chosen for additional characterization, including functional studies. Western blot analysis of SmCD59.1 and SmCD59.2 at different life cycle stages confirms that protein expression of these genes also increases during the transition to the mammalian stage. This coincides with the transition of the parasite from a complement-sensitive state to one of resistance [5]. Confocal microscopy immunolocalization studies using anti-rSmCD59.1 and anti-rSmCD59.2 antibodies confirmed previous data obtained by PiPL-C shaving showing that the proteins were surface-exposed on the tegument. On the other hand, it was clear that the proteins were also present in considerable amounts inside the parasite, which is in accord with the Western blot experiments on tegument extracts and stripped worms. On the whole, these results further support the concept that these proteins are probably involved in aspects of non-parasitic schistosome biology.

Due to the sequence similarity, we also investigated whether polyclonal antibodies directed to human CD59 (Abnova) could recognize SmCD59.1 and SmCD59.2 and whether rat anti-rSmC59.1 and anti-rSmCD59.2 polyclonal antibodies recognize human CD59 (Abnova). However, no cross reactivity could be observed in any of these studies (data not shown).

To demonstrate whether SmCD59.1 and SmCD59.2 are functional homologues of human CD59, soluble rSmCD59 proteins produced in *P. pastoris* and *E. coli* were used to examine their complement-inhibitory activity in an *in vitro* hemolytic assay designed to favor the activation of the Alternative (rabbit erythrocytes) or the Classical (antibody-sensitized sheep erythrocytes) Pathways, respectively. Soluble forms of CD59 lacking the GPI-anchor domain have been expressed in mammalian cells, insect cells and yeast and shown to have MAC-inhibitory activity in vitro [57], [61], [62], indicating that glycosylation by *Pichia pastoris* does not inhibit the protein function. However, in our study no inhibition of erythrocyte lysis was observed when NHS was pre-incubated with rSmCD59 proteins.

Membrane-targeted forms of CD59 have been shown to be more potent in inhibiting complement than the soluble forms [54], [59]. Thus, as an alternative approach, SmCD59.1 and 2 were expressed as membrane proteins (complete coding region) in CHO cells followed by treatment of transfected cells with human complement. Membrane protein expression in CHO cells may also contribute to produce rSmCD59 proteins as close to the native state as possible, which is essential for functional studies. In addition, eukaryotic cells transfected with complement regulatory proteins represent a more physiologically relevant target for *in vitro* complement experiments as compared to erythrocytes pre-treated with these proteins [63]. Despite our results showing proper localization of rSmCD59.1 and rSmCD59.2 at the plasma membrane and proper GPI-anchorage of rSmCD59.1, transfected CHO cells were not resistant to killing by human complement. Taken together, these functional analyses suggest that SmCD59.1 and SmCD59.2 do not possess inherent complement inhibitory activities.

In a third attempt to investigate the potential of SmCD59.1 and 2 to protect schistosomes from complement attack, the parasites were treated with target-specific siRNAs to induce gene expression knockdown for both targets simultaneously. Despite successful suppression of SmCD59.1 and 2 transcription and translation in schistosomula, these parasites did not become more susceptible to complement killing, by either the Alternative or the Classical Pathways as compared to control parasites. We believe that these results, together with the other complement assays and the sequence and structural comparison, strongly support the conclusion that SmCD59.1 and 2 do not have a complement regulatory function.

These schistosome CD59-like proteins probably have another function in the parasite that is unrelated to complement evasion, although it is quite likely that they are involved in some kind of molecular interaction. This hypothesis is acceptable since TFPDs commonly bind molecules, either as ligands (e.g. toxins) or membrane-attached receptors, like CD59 or urokinase/plasminogen activator receptor, uPAR [33]. Gathering all the evidence, we conclude that these CD59-like proteins do not have a complement regulatory role in schistosomes. Thus, it would be more appropriate to rename this class of proteins. However, since several papers have been published on this gene family as SmCD59 and the protein function is still unknown, it is reasonable to change the family name only when their function is elucidated. Further studies should focus on resolving the 3-D structure of the proteins and deriving their function within the biological context of the host/parasite relationship.

## Supporting Information

Figure S1 **Expression and purification of rSmCD59.1 and rSmCD59.2.** (A) SDS–PAGE (15%) analysis of pooled fractions of the recombinant proteins rSmCD59.1 and rSmCD59.2 after purification through Ni+2-charged column chromatography. Lane 1- rSmCD59.2 expressed in *E. coli*; lane 2 – rSmCD59.2 expressed in *Pichia pastoris*; lane 3 – rSmCD59.1 expressed in *Pichia pastoris*. (B) SDS-PAGE of rSmCD59.1 and rSmCD59.2 stained with Schiff's reagent to reveal the presence of glycans, (C) the same gel was stained with Coomassie to show the corresponding proteins. Positions of molecular mass standards (kDa) are indicated, 20 µg of each protein was loaded in each lane, rSmVAL4 was used as a positive glycosylated protein and BSA was used as a negative control (non-glycosylated protein).
(PDF)

Figure S2 **Phylogenetic analysis performed with protein sequences showing the relation between SmCD59 and orthologs from other Platyhelminthes species.** The sequences abbreviation are: *Schistosoma mansoni* (SmCD59.1-7), *Schistosoma japonicum* (Sj1, Sj2.1, Sj2.2, Sj2.3, Sj3, Sj4.1, Sj4.2, Sj4.3 and Sj6), *Schistosoma hematobium* (Sh1-3 and Sh5-7), *Clonorchis sinensis* (Cs-757, Cs-8328, Cs-8627 and Cs-110927), *Opisthorchis viverrini* (Ov-8524, Ov-3995, Ov-6738 and Ov-31372), *Fasciola hepatica* (Fh-6273), *Fasciola gigantica* (Fg-25430, Fg-15245 and Fg-20490), *Schmidtea mediterranea* (Smed), *Equus caballus* (Ec-Ly6), *Pongo abelii* (Pa-Ly6 and Pa-CD59), *Macaca mulatta* (Mam-Ly6), *Mus musculus* (Mm-Ly6 and Mm-CD59a), *Monodelphis domestica* (Md-Ly6), *Ornithorhynchus anatinus* (Oa-Ly6), *Homo sapiens* (Hs-Ly6 and Hs-CD59), *Rattus norvegicus* (Rn-CD59) (the accession numbers are listed in the supplementary Table S2).
(TIF)

Figure S3 **Analysis of gene expression of SmCD59.1, SmCD59.2, SmCD59.3, SmCD59.4, SmCD59.5 and SmCD59.6 genes in the egg, miracidia, cercariae, schistosomula and adult stages.** Total RNA from the different life-stages were transcribed to cDNA and analyzed by real-time RT-PCR to quantify the differences in expression levels of the genes between stages. The alfa-tubulin house-keeping control gene was used as normalizer and data were calculated according to the relative 2^−ΔΔCt^ method and shown as relative mRNA expression in relation to the stage with less expression. The data are the means (+) maximum expression variation of triplicates from the same biological sample. ND – gene expression not detected.
(PDF)

Figure S4 **Flow cytometer and fluorescent microscopy analysis of transiently transfected CHO cells for surface expression of SmCD59.1.** (A) Live cells were stained with a rat polyclonal serum anti-rSmCD59.1 followed by FITC-conjugated goat anti-rat IgG. SmCD59.1 transfected cells (red histogram in left panel) were 18.5% positive (middle panel) after subtraction of background fluorescence of cells transfected with empty vector (black histogram in left panel). Fluorescent microscopy of SmCD59.1 transfected cells (200×) shows typical membrane fluorescence pattern (right panel). (B) Similar to (A), but using CHO cells expressing hCD59 labeled with a rat MAb anti-hCD59 as positive control. Cells were 28.8% positive after background subtraction from cells transfected with empty vector. (C) To confirm GPI-anchor expression of SmCD59.1 in the cell surface, CHO cells were treated (+) or not (−) with PiPL-C and stained with anti-SmCD59.1 antibody. Percentage of fluorescent cells was determined for both samples (top panels) after background subtraction of cells transfected with empty vector. In bottom panels, CHO cells transfected with hCD59 were tested as positive control.
(PDF)

Table S1 **Set of primers/probes used to detect gene expression of SmCD59.1-7 by Real Time RT-PCR and synthetic genes used in this study.** ^a^Redesigned sequence using DNA2.0 codon optimization algorithms for expression in *Pichia pastoris*. ^b^Redesigned sequence using codon optimization algorithms for expression in mammalian cells.
(PDF)

Table S2 **List of organisms, abbreviations for gene names and accession numbers used in this study.** Databases:^*^ http://schistodb.net/schisto/, ^**^ http://bioinfosecond.vet.unimelb.edu.au/ and ^***^ http://smedgd.neuro.utah.edu/blast.php
(PDF)

## References

[1] WHO (2002) TDR Strategic Direction for Research: Schistosomiasis. World Health Organization, Geneve.

[2] Skelly PJ, Wilson RA (2006) Making sense of the schistosome surface. Adv Parasitol 63: 185–284. 17134654 10.1016/S0065-308X(06)63003-0

[3] McLaren DJ, Incani RN (1982) Schistosoma mansoni: acquired resistance of developing schistosomula to immune attack in vitro. Exp Parasitol 53: 285–298. 7060708 10.1016/0014-4894(82)90071-6

[4] Tavares CA, Cordeiro MN, Mota-Santos TA, Gazzinelli G (1980) Artificially transformed schistosomula of Schistosoma mansoni: mechanism of acquisition of protection against antibody-mediated killing. Parasitology 80: 95–104. 6992062 10.1017/s0031182000000548

[5] Marikovsky M, Levi-Schaffer F, Arnon R, Fishelson Z (1986) Schistosoma mansoni: killing of transformed schistosomula by the alternative pathway of human complement. Exp Parasitol 61: 86–94. 3943595 10.1016/0014-4894(86)90138-4

[6] Santoro F, Lachmann PJ, Capron A, Capron M (1979) Activation of complement by Schistosoma mansoni schistosomula: killing of parasites by the alternative pathway and requirement of IgG for classical pathway activation. J Immunol 123: 1551–1557. 113459

[7] Bickle QD, Ford MJ (1982) Studies on the surface antigenicity and susceptibility to antibody-dependent killing of developing schistosomula using sera from chronically infected mice and mice vaccinated with irradiated cercariae. J Immunol 128: 2101–2106. 7061855

[8] Payares G, McLaren DJ, Evans WH, Smithers SR (1985) Antigenicity and immunogenicity of the tegumental outer membrane of adult Schistosoma mansoni. Parasite Immunol 7: 45–61. 2581216 10.1111/j.1365-3024.1985.tb00478.x

[9] Klabunde J, Berger J, Jensenius JC, Klinkert MQ, Zelck UE, et al (2000) Schistosoma mansoni: adhesion of mannan-binding lectin to surface glycoproteins of cercariae and adult worms. Exp Parasitol 95: 231–239. 11038306 10.1006/expr.2000.4539

[10] Skelly PJ, Alan Wilson R (2006) Making sense of the schistosome surface. Adv Parasitol 63: 185–284. 17134654 10.1016/S0065-308X(06)63003-0

[11] Braschi S, Curwen RS, Ashton PD, Verjovski-Almeida S, Wilson A (2006) The tegument surface membranes of the human blood parasite Schistosoma mansoni: a proteomic analysis after differential extraction. Proteomics 6: 1471–1482. 16447162 10.1002/pmic.200500368

[12] Braschi S, Wilson RA (2006) Proteins exposed at the adult schistosome surface revealed by biotinylation. Mol Cell Proteomics 5: 347–356. 16269422 10.1074/mcp.M500287-MCP200

[13] Castro-Borges W, Dowle A, Curwen RS, Thomas-Oates J, Wilson RA (2011) Enzymatic shaving of the tegument surface of live schistosomes for proteomic analysis: a rational approach to select vaccine candidates. PLoS Negl Trop Dis 5: e993. 21468311 10.1371/journal.pntd.0000993 PMC3066142

[14] Abath FG, Werkhauser RC (1996) The tegument of Schistosoma mansoni: functional and immunological features. Parasite Immunol 18: 15–20. 9223152 10.1046/j.1365-3024.1996.d01-6.x

[15] Ghendler Y, Parizade M, Arnon R, McKerrow JH, Fishelson Z (1996) Schistosoma mansoni: evidence for a 28-kDa membrane-anchored protease on schistosomula. Exp Parasitol 83: 73–82. 8654554 10.1006/expr.1996.0051

[16] Marikovsky M, Arnon R, Fishelson Z (1988) Proteases secreted by transforming schistosomula of Schistosoma mansoni promote resistance to killing by complement. J Immunol 141: 273–278. 3132503

[17] Schroeder H, Skelly PJ, Zipfel PF, Losson B, Vanderplasschen A (2009) Subversion of complement by hematophagous parasites. Dev Comp Immunol 33: 5–13. 18762211 10.1016/j.dci.2008.07.010 PMC2642905

[18] Skelly PJ (2004) Intravascular schistosomes and complement. Trends Parasitol 20: 370–374. 15246320 10.1016/j.pt.2004.05.007

[19] Laclette JP, Shoemaker CB, Richter D, Arcos L, Pante N, et al (1992) Paramyosin inhibits complement C1. J Immunol 148: 124–128. 1727860

[20] van Dam GJ, Seino J, Rotmans JP, Daha MR, Deelder AM (1993) Schistosoma mansoni circulating anodic antigen but not circulating cathodic antigen interacts with complement component C1q. Eur J Immunol 23: 2807–2812. 8223856 10.1002/eji.1830231113

[21] Silva EE, Clarke MW, Podesta RB (1993) Characterization of a C3 receptor on the envelope of Schistosoma mansoni. J Immunol 151: 7057–7066. 8258710

[22] Horta MF, Ramalho-Pinto FJ (1991) Role of human decay-accelerating factor in the evasion of Schistosoma mansoni from the complement-mediated killing in vitro. J Exp Med 174: 1399–1406. 1720809 10.1084/jem.174.6.1399 PMC2119036

[23] Pearce EJ, Hall BF, Sher A (1990) Host-specific evasion of the alternative complement pathway by schistosomes correlates with the presence of a phospholipase C-sensitive surface molecule resembling human decay accelerating factor. J Immunol 144: 2751–2756. 1690776

[24] Parizade M, Arnon R, Lachmann PJ, Fishelson Z (1994) Functional and antigenic similarities between a 94-kD protein of Schistosoma mansoni (SCIP-1) and human CD59. J Exp Med 179: 1625–1636. 7513011 10.1084/jem.179.5.1625 PMC2191495

[25] Wilson RA (2012) Proteomics at the schistosome-mammalian host interface: any prospects for diagnostics or vaccines? Parasitology 139: 1178–1194. 22717150 10.1017/S0031182012000339

[26] Ninomiya H, Stewart BH, Rollins SA, Zhao J, Bothwell AL, et al (1992) Contribution of the N-linked carbohydrate of erythrocyte antigen CD59 to its complement-inhibitory activity. J Biol Chem 267: 8404–8410. 1373727

[27] Deng J, Gold D, LoVerde PT, Fishelson Z (2003) Inhibition of the complement membrane attack complex by Schistosoma mansoni paramyosin. Infect Immun 71: 6402–6410. 14573661 10.1128/IAI.71.11.6402-6410.2003 PMC219572

[28] Wilson RA, Coulson PS (2009) Immune effector mechanisms against schistosomiasis: looking for a chink in the parasite's armour. Trends Parasitol 25: 423–431. 19717340 10.1016/j.pt.2009.05.011 PMC3686490

[29] Farias LP, Tararam CA, Miyasato PA, Nishiyama MY Jr, Oliveira KC, et al (2010) Screening the Schistosoma mansoni transcriptome for genes differentially expressed in the schistosomulum stage in search for vaccine candidates. Parasitol Res 108: 123–135. 20852890 10.1007/s00436-010-2045-1

[30] Galat A (2008) The three-fingered protein domain of the human genome. Cell Mol Life Sci 65: 3481–3493. 18821057 10.1007/s00018-008-8473-8 PMC11131612

[31] McKenzie IF, Gardiner J, Cherry M, Snell GD (1977) Lymphocyte antigens: Ly-4, Ly-6, and Ly-7. Transplant Proc 9: 667–669. 68598

[32] Gumley TP, McKenzie IF, Sandrin MS (1995) Tissue expression, structure and function of the murine Ly-6 family of molecules. Immunol Cell Biol 73: 277–296. 7493764 10.1038/icb.1995.45

[33] Galat A, Gross G, Drevet P, Sato A, Menez A (2008) Conserved structural determinants in three-fingered protein domains. FEBS J 275: 3207–3225. 18485004 10.1111/j.1742-4658.2008.06473.x

[34] Dalton JP, Day SR, Drew AC, Brindley PJ (1997) A method for the isolation of schistosome eggs and miracidia free of contaminating host tissues. Parasitology 115 ((Pt 1)) 29–32. 9226954 10.1017/s0031182097001091

[35] Basch PF (1981) Cultivation of Schistosoma mansoni in vitro. I. Establishment of cultures from cercariae and development until pairing. J Parasitol 67: 179–185. 7241277

[36] Petersen TN, Brunak S, von Heijne G, Nielsen H (2011) SignalP 4.0: discriminating signal peptides from transmembrane regions. Nat Methods 8: 785–786. 21959131 10.1038/nmeth.1701

[37] Eisenhaber B, Bork P, Eisenhaber F (1999) Prediction of potential GPI-modification sites in proprotein sequences. J Mol Biol 292: 741–758. 10497036 10.1006/jmbi.1999.3069

[38] Marti-Renom MA, Stuart AC, Fiser A, Sanchez R, Melo F, et al (2000) Comparative protein structure modeling of genes and genomes. Annu Rev Biophys Biomol Struct 29: 291–325. 10940251 10.1146/annurev.biophys.29.1.291

[39] Laskowski RA, MacArthur MW, Moss DS, Thornton JM (1993) PROCHECK: a program to check the stereochemical quality of protein structures. Journal of Applied Crystallography 26: 283–291.

[40] Page RD (1996) TreeView: an application to display phylogenetic trees on personal computers. Comput Appl Biosci 12: 357–358. 8902363 10.1093/bioinformatics/12.4.357

[41] Rofatto HK, Tararam CA, Borges WC, Wilson RA, Leite LC, et al (2009) Characterization of phosphodiesterase-5 as a surface protein in the tegument of Schistosoma mansoni. Mol Biochem Parasitol 166: 32–41. 19428670 10.1016/j.molbiopara.2009.02.006

[42] Livak KJ, Schmittgen TD (2001) Analysis of relative gene expression data using real-time quantitative PCR and the 2(-Delta Delta C(T)) Method. Methods 25: 402–408. 11846609 10.1006/meth.2001.1262

[43] Krautz-Peterson G, Camargo S, Huggel K, Verrey F, Shoemaker CB, et al (2007) Amino acid transport in schistosomes: Characterization of the permeaseheavy chain SPRM1hc. J Biol Chem 282: 21767–21775. 17545149 10.1074/jbc.M703512200

[44] Ramos CR, Abreu PA, Nascimento AL, Ho PL (2004) A high-copy T7 Escherichia coli expression vector for the production of recombinant proteins with a minimal N-terminal His-tagged fusion peptide. Braz J Med Biol Res 37: 1103–1109. 15273812 10.1590/s0100-879x2004000800001

[45] Farias LP, Rodrigues D, Cunna V, Rofatto HK, Faquim-Mauro EL, et al (2012) Schistosoma mansoni venom allergen like proteins present differential allergic responses in a murine model of airway inflammation. PLoS Negl Trop Dis 6: e1510. 22347513 10.1371/journal.pntd.0001510 PMC3274501

[46] Roberts SM, MacGregor AN, Vojvodic M, Wells E, Crabtree JE, et al (1983) Tegument surface membranes of adult Schistosoma mansoni: development of a method for their isolation. Mol Biochem Parasitol 9: 105–127. 6669162 10.1016/0166-6851(83)90104-4

[47] Farias LP, Cardoso FC, Miyasato PA, Montoya BO, Tararam CA, et al (2010) Schistosoma mansoni Stomatin like protein-2 is located in the tegument and induces partial protection against challenge infection. PLoS Negl Trop Dis 4: e597. 20161725 10.1371/journal.pntd.0000597 PMC2817717

[48] Mair GR, Maule AG, Day TA, Halton DW (2000) A confocal microscopical study of the musculature of adult Schistosoma mansoni. Parasitology 121 ((Pt 2)) 163–170. 11085236 10.1017/s0031182099006174

[49] Amano MT, Ferriani VP, Florido MP, Reis ES, Delcolli MI, et al (2008) Genetic analysis of complement C1s deficiency associated with systemic lupus erythematosus highlights alternative splicing of normal C1s gene. Mol Immunol 45: 1693–1702. 18062908 10.1016/j.molimm.2007.09.034

[50] Servais G, Walmagh J, Duchateau J (1991) Simple quantitative haemolytic microassay for determination of complement alternative pathway activation (AP50). J Immunol Methods 140: 93–100. 2061618 10.1016/0022-1759(91)90130-8

[51] Zhao J, Rollins SA, Maher SE, Bothwell AL, Sims PJ (1991) Amplified gene expression in CD59-transfected Chinese hamster ovary cells confers protection against the membrane attack complex of human complement. J Biol Chem 266: 13418–13422. 1712784

[52] Huang Y, Smith CA, Song H, Morgan BP, Abagyan R, et al (2005) Insights into the human CD59 complement binding interface toward engineering new therapeutics. J Biol Chem 280: 34073–34079. 16079145 10.1074/jbc.M504922200

[53] Krautz-Peterson G, Radwanska M, Ndegwa D, Shoemaker CB, Skelly PJ (2007) Optimizing gene suppression in schistosomes using RNA interference. Mol Biochem Parasitol 153: 194–202. 17420062 10.1016/j.molbiopara.2007.03.006

[54] Jones JT, Helm CN, Kusel JR (1988) Variation in susceptibility of Schistosoma mansoni to damage by polycations. Mol Biochem Parasitol 30: 35–44. 2456464 10.1016/0166-6851(88)90130-2

[55] Bodian DL, Davis SJ, Morgan BP, Rushmere NK (1997) Mutational analysis of the active site and antibody epitopes of the complement-inhibitory glycoprotein, CD59. J Exp Med 185: 507–516. 9053451 10.1084/jem.185.3.507 PMC2196035

[56] Song H, He C, Knaak C, Guthridge JM, Holers VM, et al (2003) Complement receptor 2-mediated targeting of complement inhibitors to sites of complement activation. J Clin Invest 111: 1875–1885. 12813023 10.1172/JCI17348 PMC161422

[57] Sugita Y, Ito K, Shiozuka K, Suzuki H, Gushima H, et al (1994) Recombinant soluble CD59 inhibits reactive haemolysis with complement. Immunology 82: 34–41. 7519172 PMC1414854

[58] Marchler-Bauer A, Lu S, Anderson JB, Chitsaz F, Derbyshire MK, et al (2011) CDD: a Conserved Domain Database for the functional annotation of proteins. Nucleic Acids Res 2011 Jan;39 ((Database issue)): D225–9 Epub 2010 Nov 24. 10.1093/nar/gkq1189 PMC3013737 21109532

[59] Liu G, Zhang J, Chen X (2007) Molecular and functional characterization of a CD59 analogue from large yellow croaker Pseudosciana crocea. Mol Immunol 44: 3661–3671. 17531319 10.1016/j.molimm.2007.04.006

[60] Parker-Manuel SJ, Ivens AC, Dillon GP, Wilson RA (2011) Gene expression patterns in larval Schistosoma mansoni associated with infection of the mammalian host. PLoS Negl Trop Dis 5: e1274. 21912711 10.1371/journal.pntd.0001274 PMC3166049

[61] Meri S, Lehto T, Sutton CW, Tyynela J, Baumann M (1996) Structural composition and functional characterization of soluble CD59: heterogeneity of the oligosaccharide and glycophosphoinositol (GPI) anchor revealed by laser-desorption mass spectrometric analysis. Biochem J 316 ((Pt 3)) 923–935. 8670172 10.1042/bj3160923 PMC1217438

[62] Quigg RJ, He C, Hack BK, Alexander JJ, Morgan BP (2000) Production and functional analysis of rat CD59 and chimeric CD59-Crry as active soluble proteins in Pichia pastoris. Immunology 99: 46–53. 10651940 10.1046/j.1365-2567.2000.00945.x PMC2327136

[63] Huang J, Gou D, Zhen C, Jiang D, Mao X, et al (2001) Protection of xenogeneic cells from human complement-mediated lysis by the expression of human DAF, CD59 and MCP. FEMS Immunol Med Microbiol 31: 203–209. 11720816 10.1111/j.1574-695X.2001.tb00521.x

