# Supplementary figures and images for: On the Three-Finger Protein Domain Fold and CD59-Like Proteins in Schistosoma mansoni
Source: PLoS Negl Trop Dis. 2013 Oct 24;7(10):e2482. doi: 10.1371/journal.pntd.0002482 (PMC3812095; doi:10.1371/journal.pntd.0002482)

**A**

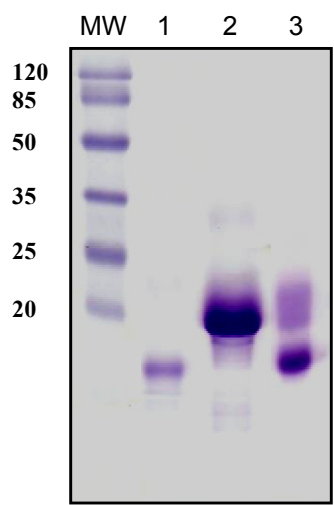

**B**

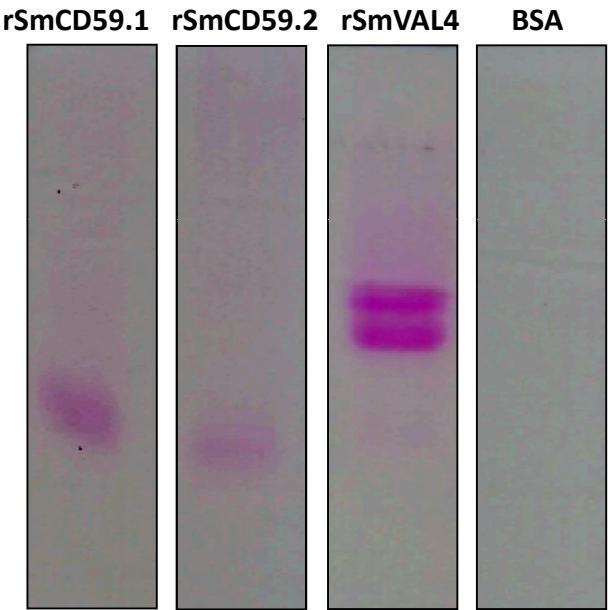

**C**

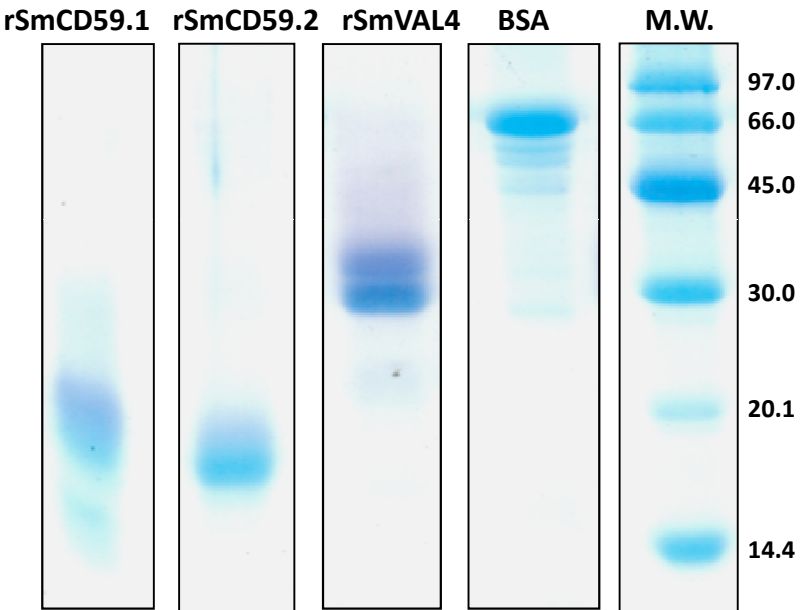

Supplement: Figure S1 — Expression and purification of rSmCD59.1 and rSmCD59.2. (A) SDS–PAGE (15%) analysis of pooled fractions of the recombinant proteins rSmCD59.1 and rSmCD59.2 after purification through Ni+2-charged column chromatography. Lane 1- rSmCD59.2 expressed in E. coli; lane 2 – rSmCD59.2 expressed in Pichia pastoris; lane 3 – rSmCD59.1 expressed in Pichia pastoris. (B) SDS-PAGE of rSmCD59.1 and rSmCD59.2 stained with Schiff's reagent to reveal the presence of glycans, (C) the same gel was stained with Coomassie to show the corresponding proteins. Positions of molecular mass standards (kDa) are indicated, 20 µg of each protein was loaded in each lane, rSmVAL4 was used as a positive glycosylated protein and BSA was used as a negative control (non-glycosylated protein). (PDF) [file pntd.0002482.s001.pdf]

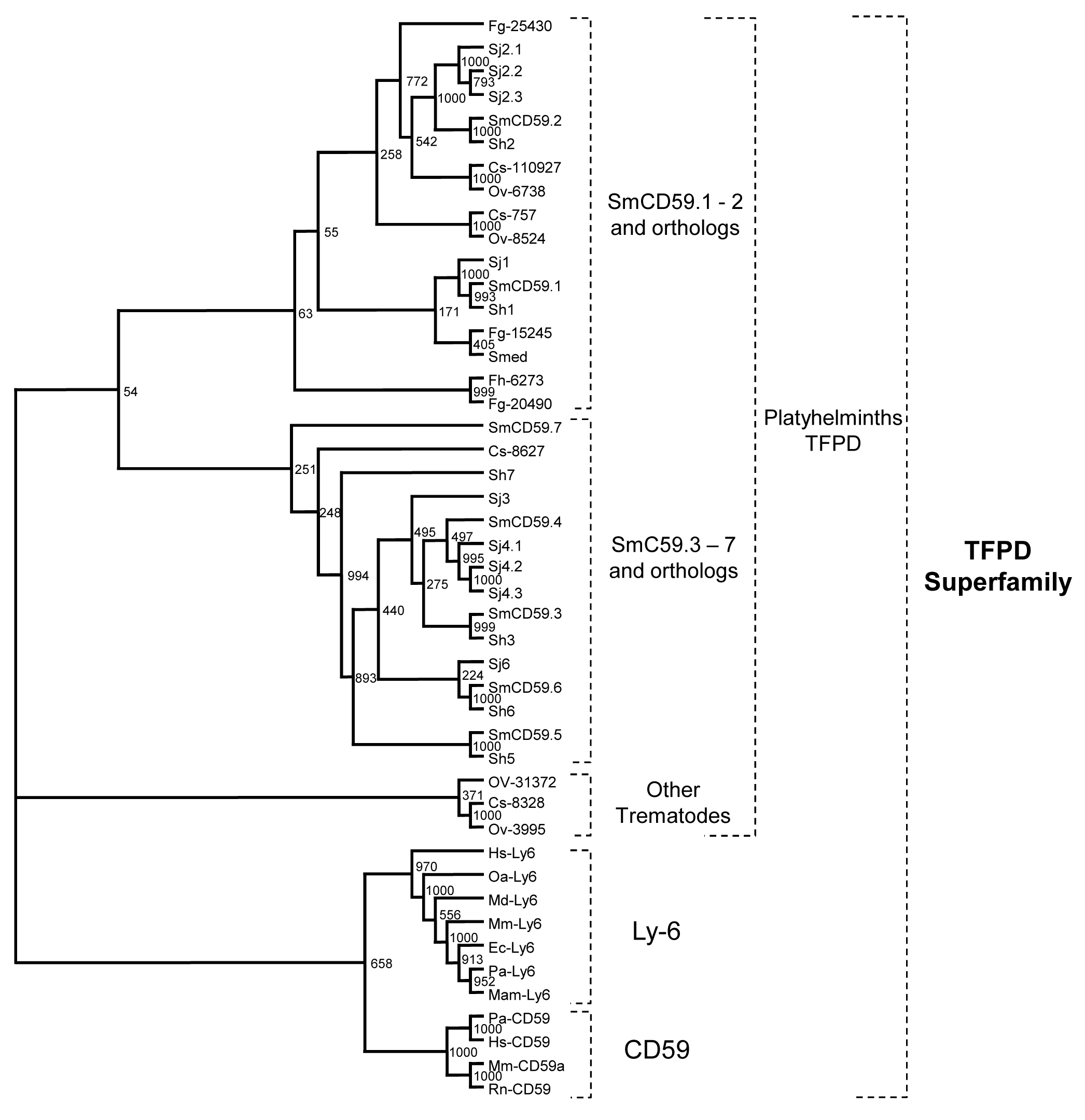

Supplement: Figure S2 — Phylogenetic analysis performed with protein sequences showing the relation between SmCD59 and orthologs from other Platyhelminthes species. The sequences abbreviation are: Schistosoma mansoni (SmCD59.1-7), Schistosoma japonicum (Sj1, Sj2.1, Sj2.2, Sj2.3, Sj3, Sj4.1, Sj4.2, Sj4.3 and Sj6), Schistosoma hematobium (Sh1-3 and Sh5-7), Clonorchis sinensis (Cs-757, Cs-8328, Cs-8627 and Cs-110927), Opisthorchis viverrini (Ov-8524, Ov-3995, Ov-6738 and Ov-31372), Fasciola hepatica (Fh-6273), Fasciola gigantica (Fg-25430, Fg-15245 and Fg-20490), Schmidtea mediterranea (Smed), Equus caballus (Ec-Ly6), Pongo abelii (Pa-Ly6 and Pa-CD59), Macaca mulatta (Mam-Ly6), Mus musculus (Mm-Ly6 and Mm-CD59a), Monodelphis domestica (Md-Ly6), Ornithorhynchus anatinus (Oa-Ly6), Homo sapiens (Hs-Ly6 and Hs-CD59), Rattus norvegicus (Rn-CD59) (the accession numbers are listed in the supplementary Table S2). (TIF) [file pntd.0002482.s002.tif]

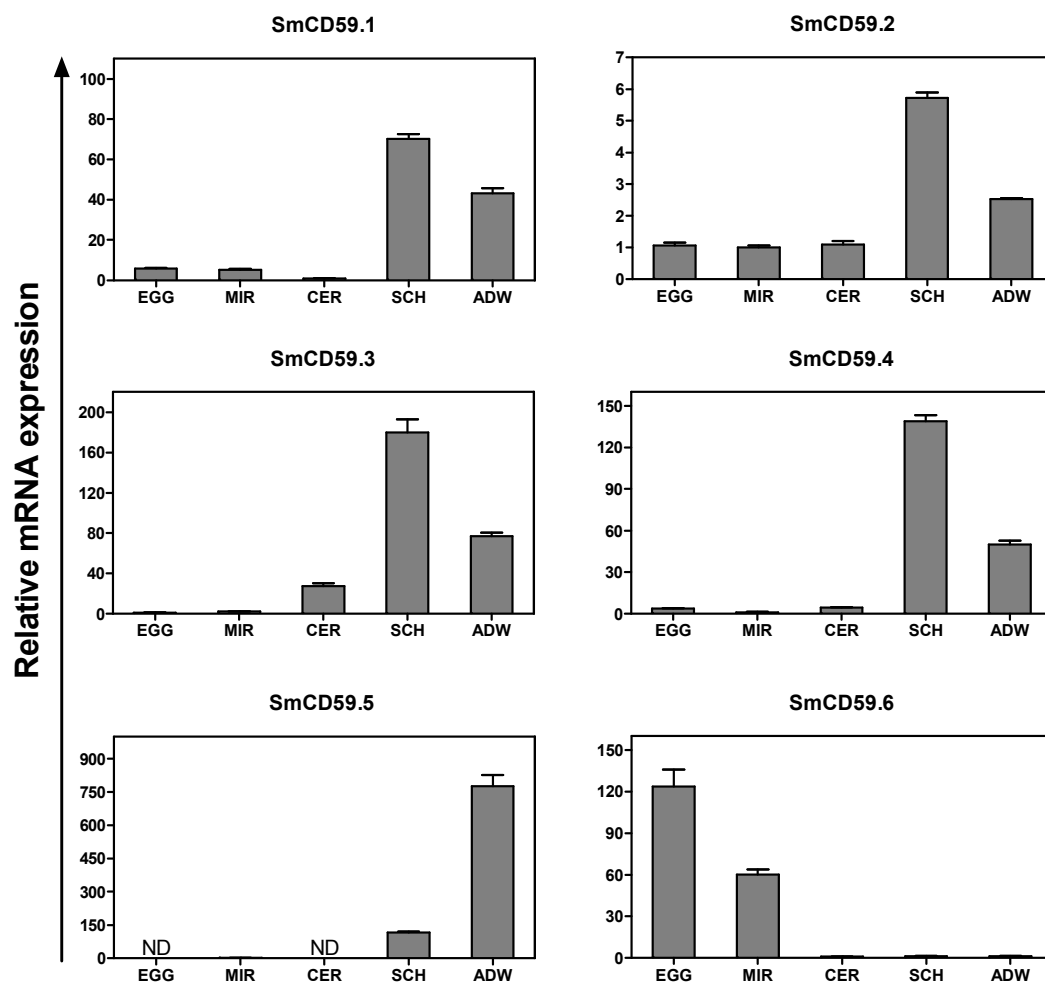

Supplement: Figure S3 — Analysis of gene expression of SmCD59.1, SmCD59.2, SmCD59.3, SmCD59.4, SmCD59.5 and SmCD59.6 genes in the egg, miracidia, cercariae, schistosomula and adult stages. Total RNA from the different life-stages were transcribed to cDNA and analyzed by real-time RT-PCR to quantify the differences in expression levels of the genes between stages. The alfa-tubulin house-keeping control gene was used as normalizer and data were calculated according to the relative 2−ΔΔCt method and shown as relative mRNA expression in relation to the stage with less expression. The data are the means (+) maximum expression variation of triplicates from the same biological sample. ND – gene expression not detected. (PDF) [file pntd.0002482.s003.pdf]

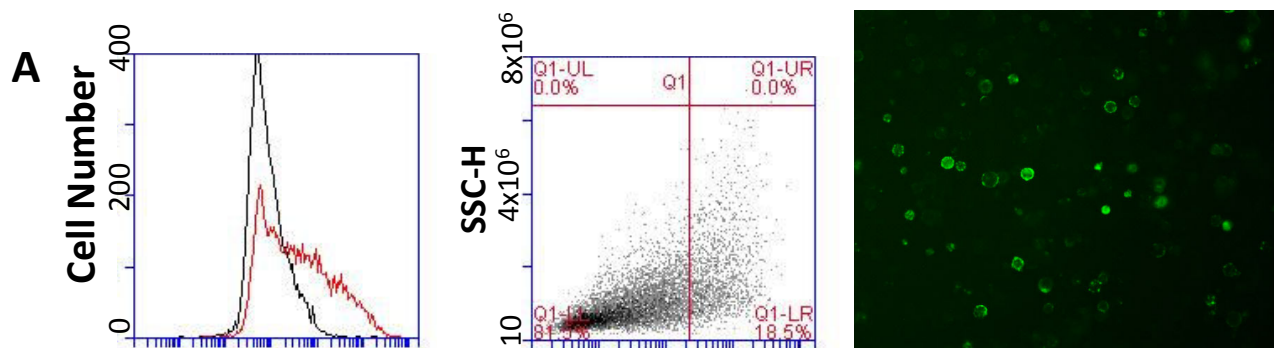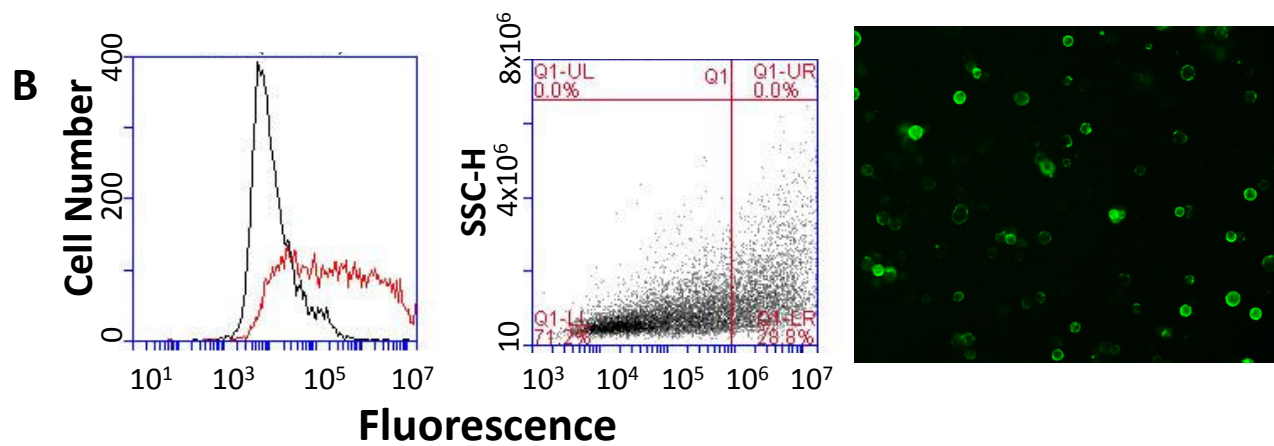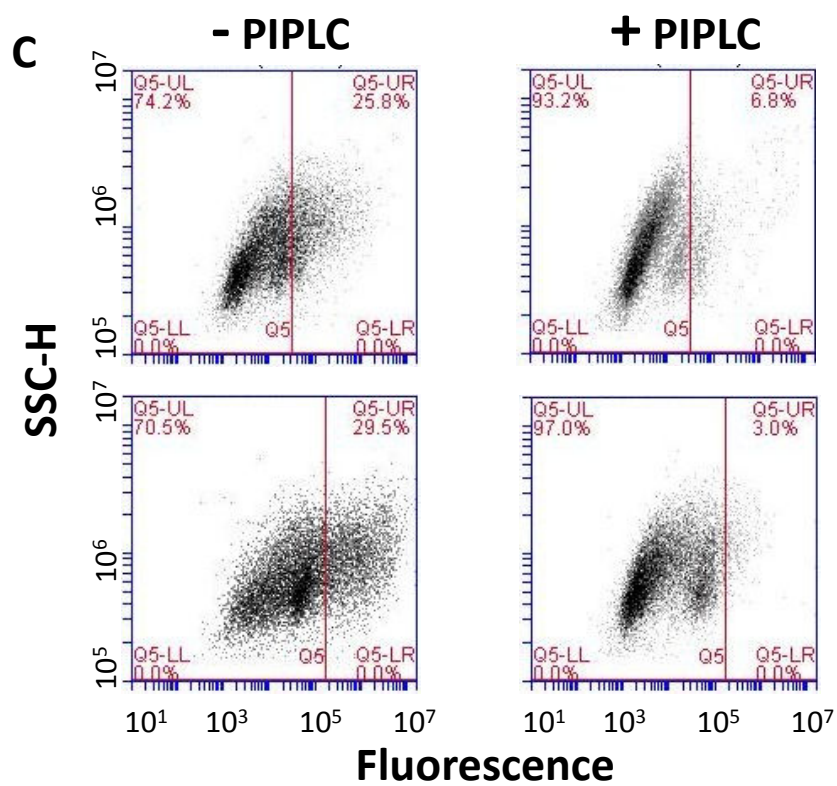

Supplement: Figure S4 — Flow cytometer and fluorescent microscopy analysis of transiently transfected CHO cells for surface expression of SmCD59.1. (A) Live cells were stained with a rat polyclonal serum anti-rSmCD59.1 followed by FITC-conjugated goat anti-rat IgG. SmCD59.1 transfected cells (red histogram in left panel) were 18.5% positive (middle panel) after subtraction of background fluorescence of cells transfected with empty vector (black histogram in left panel). Fluorescent microscopy of SmCD59.1 transfected cells (200×) shows typical membrane fluorescence pattern (right panel). (B) Similar to (A), but using CHO cells expressing hCD59 labeled with a rat MAb anti-hCD59 as positive control. Cells were 28.8% positive after background subtraction from cells transfected with empty vector. (C) To confirm GPI-anchor expression of SmCD59.1 in the cell surface, CHO cells were treated (+) or not (−) with PiPL-C and stained with anti-SmCD59.1 antibody. Percentage of fluorescent cells was determined for both samples (top panels) after background subtraction of cells transfected with empty vector. In bottom panels, CHO cells transfected with hCD59 were tested as positive control. (PDF) [file pntd.0002482.s004.pdf]
