# Supplementary material for: On the Three-Finger Protein Domain Fold and CD59-Like Proteins in Schistosoma mansoni
Source: PLoS Negl Trop Dis. 2013 Oct 24;7(10):e2482. doi: 10.1371/journal.pntd.0002482 (PMC3812095; doi:10.1371/journal.pntd.0002482)
Supplement: Table S1 — Set of primers/probes used to detect gene expression of SmCD59.1-7 by Real Time RT-PCR and synthetic genes used in this study. aRedesigned sequence using DNA2.0 codon optimization algorithms for expression in Pichia pastoris. bRedesigned sequence using codon optimization algorithms for expression in mammalian cells. (PDF) [file pntd.0002482.s005.pdf]

Table S1. Set of primers/probes used to detect gene expression of SmCD59.1-7 by qRT-PCR and synthetic genes used in this study.

TaqMan System

SmCD59	*S. mansoni* Gene DB	Forward primer (5'-3')	Reverse Primer (5'-3')	FAM-probes (5'-3')
1	Smp_019350	TGTTACGTCTCTTC<br>CCCATTCTACT	GGGTCTTGACACTTACTAC<br>ACACAT	ACGATGGCCTAAACCTGT
2	Smp_105220	GATTGTCCTAATCCATT<br>CGATAAGACACA	CTTGCTACACAATCTTTCGC<br>AATTCTATAATT	ACTGTTCGACAAAAGTTAC
3	Smp_081900	ACAGATTCTCCTTTTGA<br>TCTTGCTGAT	TCGAATGGATCTGGACAAT<br>TTAAGCA	TCTACATTGCAGTTTCTCC
4	Smp_166340	CGTCGTCACCACTAGTA<br>CACAATTA	TGTACATTGTCTGATCGGG<br>TTTGAA	ATGGTGTGCAAAACTT
5	Smp_081920	CCACACTCACTACTAGT<br>TAATGAACAAGAT	ACAGAGTCTAAATGGGTAG<br>GGTACA	ATGGTGTGCAAAAATT
6	Smp_166350	AGTAGGCAACCAAAGT<br>GGATGTG	TGAACATTTTCTTATTGGAG<br>TACGACGAT	ATGGTGTGTCAAGATTAT
7	Smp_125250	CAGGGAAAGCCAACTT<br>CACAATT	GTTAGACAGCTCCTTGTTGT<br>AGAGT	CTGCCAGAAAATAGTTAGA<br>CTGA
SmTPI	Smp_003990	CATACTTGGACATTCTG<br>AGCGTAGA	ACCTTCAGCAAGTGCATGT<br>TGA	CAATAAGTTCATCAGATTCA<br>C

Sybr System

SmCD59	*S. mansoni* Gene DB	Forward primer (5'-3')	Reverse Primer (5'-3')
1	Smp_019350	GTCAACAGGTTTAGGCCATCGT	CTTATTGTTGAACAGAAGGTACAACCA
2	Smp_105220	GAATTGCGAAAGATTGTGTAGCA	CACCACGACGATCTTGTGGA
3	Smp_081900	GCTGCTATGAATGCTTAAATTGTCC	CATAAGCTTTGCACACCACACAC
4	Smp_166340	TGGTGTGCAAAACTTGAAGTTCC	TGACAGCAATAATATCTAAGCTTGGG
5	Smp_081920	TGACAATGTTGCTTTTGTTGAACA	GTGAGTGTGGATTAAATGGTACTGAAC
6	Smp_166350	GCTGCTACACTTGCGAAACTTG	AATCTTGACACACCATGCACATC
α-Tubulin	Smp_090120	CCATTTATGATATTTGTCGACGGA	TTTGTGTAGGTTGGACGCTCTATATCTA

Synthetic Genes

SmCD59.2[a]	TCTAGAGCATTACAGTAATCCTCGTCGCAGCATTCAGTTACCAGTCCGGCTTTACCACCCCTTCTATCTTGGGG<br>CACACATGAAGCAACACAGTCTTTTGCGATTCTATAGTTATCCTCATCTCGATAGGTGTAAACCGTACGACAA<br>AAGTTACAGTTGCCCAATTCAGTAATCTGTGTCTTGTCGAATGGATTAGGGCAATCAGAACATCTGTAACATT<br>TGACCTTCTTATTCTTGAATTC
SmCD59.1[b]	ATGATTTACAATTTTAATGTTATGATGCTGAGACTTTTTCCTATTTTACTGCTGGTGATTTTTACTTCAACTGGA<br>CTGGGACACAGATGCTACGTGTGTTCTAAATGCCAAGATCCTTTTAGAGTTAAGGATACAGAAATACAGAAT<br>GGATGTACCTTTTGCAGTACCATAAGAACTTACGTTCAAGATAAGCTGCAGGTAACCTCAAGAAGTTGTGTTC<br>CAGTATGCGTGGAGGCTGATGCAAGAAGATCTGGATCAGGTATTGTAACTAGTTGCTGTCAAGATGATCTGT<br>GTAATTCAGGTACTCAAACACAGATTAGTATGACACTGATACTAAGTTCTCTTTTTAGTATATTACCTCTTTTAT<br>CTAGATACTTT
SmCD59.2[b]	ATGAAAGTGCTGGGAATTTGTGTGATACTGACTCTGATTTTTAATGGAATAAATTGCATAAAGAATAAGAAA<br>GTGAAGTGCTATAGATGCTCTGATTGTCCTAATCCATTTGATAAGACCCAAATTACTGAACTGGGAAATTGCA<br>ATTTTTGTAGAACAGTGTACACCTATAGAGATGAGGATAATTACAGAATTGCTAAGGATTGCGTTGCAAGTTG<br>TGTACCTCAGGATAGAAGAGGAGGAAAGGCCGGTCTTGTTACCGAATGCTGTGATGAGGATTACTGCAATG<br>CTTCACCAAAACATTATAGTATTTCTTTTCTTTTAATAACTAGTTTTACAATTTTTATAACTTACACAAATAAGTT<br>TATTTAT

[a]Redesigned sequence using DNA2.0 codon optimization algorithms for expression in *Pichia pastoris.*
[b]Redesigned sequence using codon optimization algorithms for expression in mammalian cells.
