# Supplementary material for: On the Three-Finger Protein Domain Fold and CD59-Like Proteins in Schistosoma mansoni
Source: PLoS Negl Trop Dis. 2013 Oct 24;7(10):e2482. doi: 10.1371/journal.pntd.0002482 (PMC3812095; doi:10.1371/journal.pntd.0002482)
Supplement: Table S2 — List of organisms, abbreviations for gene names and accession numbers used in this study. Databases:* http://schistodb.net/schisto/, ** http://bioinfosecond.vet.unimelb.edu.au/ and *** http://smedgd.neuro.utah.edu/blast.php (PDF) [file pntd.0002482.s006.pdf]

Table S2. List of organisms, abbreviations for gene names and accession numbers used in this study.

Organism	Abbreviation gene name	Accession number
Schistosoma mansoni	SmCD59.1	Smp_019350
	SmCD59.2	Smp_105220
	SmCD59.3	Smp_081900.2
	SmCD59.4	Smp_166340
	SmCD59.5	Smp_081920
	SmCD59.6	Smp_166350
	SmCD59.7	Smp_125250
Schistosoma japonicum	Sj1	AAW26563.1
	Sj2.1	AAW27735.1
	Sj2.2	CAX74555.1
	Sj2.3	CAX70150.1
	Sj3	AAW26917.1
	Sj4.1	CAX69649.1
	Sj4.2	CAX74000.1
	Sj4.3	CAX69837.1
	Sj6	CAX69697.1
Schistosoma hematobium	Sh1	Sha_106826*
	Sh2	Sha_200489*
	Sh3	Sha_200766*
	Sh5	Sha_300533*
	Sh6	Sha_109174*
	Sh7	Sha_109175*
Clonorchis sinensis	Cs-757	GAA51655.1
	Cs-8328	Cs_8328**
	Cs-8627	Cs_8627**
	Cs-110927	GAA56456.1
Opisthorchis viverrini	Ov-3995	OV1_c3995**
	Ov-6738	Ov_Contig6738**
	OV-8524	OV1_c8524**
	Ov-31372	OV1_c31372**
Fasciola hepatica	Fh-6273	Fh_Contig6273**
Fasciola gigantica	Fg-15245	Contig15245**
	Fg-20490	Contig20490**
	Fg-25430	Contig25430**
Schmidtea mediterranea	Smed	mk4.006032.00.01***
Equus caballus	Ec-Ly6	XP_001496782.1
Pan troglodytes	Pa-Ly6	XP_002819547.1
Macaca mulatta	Mam-Ly6	NP_001028116.1
Mus musculus	Mm-Ly6	NP_035968.1
Monodelphis domestica	Md-Ly6	XP_001381791.2
Ornithorhynchus anatinus	Oa-Ly6	XP_001512986.1
Homo sapiens	Hs-Ly6	DAAH22806.1
Saimiriine herpesvirus	Sah-CD59	CAA73629.1
Mus musculus	Mm-CD59	NP_031678.1
Rattus norvegicus	Rn-CD59	NP_037057.1
Pongo abelii	Pa-CD59	NP_001126861.1
Homo sapiens	Hs-CD59	NP_000602.1

Databases: *http://schistodb.net/schisto/, ***http://bioinfosecond.vet.unimelb.edu.au/* and ****http://smedgd.neuro.utah.edu/blast.php*
